# Hyderabad’s egg value chain: investigating potential influences on childhood stunting

**DOI:** 10.1007/s12571-025-01633-6

**Published:** 2026-01-31

**Authors:** Emma Gomez de Gracia, Barbara Häsler, Srinu Rotta, Archana Konapur, Thanammal Ravichandran, Paula Dominguez-Salas, Mathew Hennessey, Delia Randolph, Little Flower Augustine, Naveen Kumar Ramachandrappa, Claire Heffernan, Santosh Kumar Banjara, Bharati Kulkarni

**Affiliations:** 1 Veterinary Epidemiology Economics and Public Health Group, Department of Pathobiology and Population Sciences, Royal Veterinary College, London, UK; 2 ICMR-National Institute of Nutrition, DHR, Ministry of Health and Family Welfare, GoI, Beside Tarnaka metro station, Hyderabad, Telangana India; 3 Department of Social Works, Kumaraguru College of Liberal Arts and Science, Coimbatore, Tamil Nadu India; 4 Natural Resources Institute, University of Greenwich, London, UK; 5 International Livestock Research Institute, Nairobi, Kenya; 6 London International Development Centre, London School of Hygiene and Tropical Medicine, London, UK

**Keywords:** Stunting, Value chain analysis, Child malnutrition, Egg value chain, Reflexive thematic analysis, Food safety

## Abstract

In 2024, stunting affected over 150 million children under five worldwide, with India being one of the countries with the highest prevalence. The links between stunting, animal health and the health of the environment make a multi-sectoral and interdisciplinary approach essential to combatting childhood stunting in affected countries. This study examined the egg value chain in Hyderabad, India, investigating its potential influences on childhood stunting. Semi-structured interviews with ten key informants gathered data on the egg value chain’s spatial, logistical, economic, and social dimensions. The interviews were analysed through reflexive thematic analysis. A conceptual framework was developed showcasing the links between food value chains and stunting and used as a lens through which to analyse deductively the qualitative data. Reflexive thematic analysis generated themes encompassing the nutritional quality and food safety of eggs accessed by children, how seasonality affected consumption, and the lack of regulation, which forces actors in the chain to depend on trust. The incorporation of a nutrition-sensitive perspective into the value chain analysis revealed stunting-related opportunities and constraints. These findings offer a basis for dialogue with public and private stakeholders to develop targeted interventions that can support efforts to combat stunting in Hyderabad. Additionally, they underscore the method’s utility in generating a comprehensive understanding of stunting determinants and showcase the potential of value chain interventions in addressing the factors contributing to stunting.

**Supplementary information:**

The online version contains supplementary material available at 10.1007/s12571-025-01633-6.

## Introduction

Child stunting, measured through height-for-age z (HAZ) scores, is an expression of chronic malnutrition (Perumal et al., 2018) that impacts both physical and cognitive development (Sinha et al., 2018). Its multifaceted causes span contextual factors such as political economy, healthcare, and education; and household factors, including food and water availability and safety, parental care and infection, among others (WHO, 2017). Despite its rapid economic growth and agricultural success, India is one of the countries with the highest prevalence of child stunting globally, at 31.7% in 2022 (UNICEF et al., 2023). Notwithstanding rising per capita income (Kadiyala et al., 2013), the elevated prices of nutritious foods, particularly of animal source foods (ASFs) (Kadiyala et al., 2014), limit access to essential nutrients, contributing to widespread nutritional deficiencies in low-income households (Ekanayake, 2020).

The UKRI GCRF Action Against Stunting Hub (AASH), an interdisciplinary research programme working in India, Indonesia and Senegal, applied a “Whole Child Approach” to develop a holistic understanding of stunting focussing on five interconnected environments: the biological, home, educational, broader food system and shared community-level values (Jobarteh et al., 2024). The food systems workstream of the AASH focused on investigating market-driven strategies that could promote the consumption of nutrient-rich foods while considering food safety hazards (Cooper et al., 2024). A nutrition-sensitive approach through food value chain analysis (VCA) was used to understand the structures of selected value chains (VCs) for foods with potential to reduce stunting and to identify food safety risks and opportunities to alleviate stunting (Cooper et al., 2024).

Value chain analysis aligns with the necessity for a comprehensive, cross-sectoral strategy advocated by the global nutrition community (Sawadogo-Lewis et al., 2021), as it facilitates both nutrition-specific and nutrition-sensitive interventions (Ruel et al., 2013). A nutrition-sensitive VCA considers the VC of a specific food commodity to identify opportunities for interventions that can improve nutritional outcomes (De la Peña and Garret, 2018). It is particularly relevant for micronutrient-rich foods like ASFs (Headey et al., 2018) that have been suggested as a vehicle to enhance nutrient consumption in early childhood (Shapiro et al., 2019) as they have essential nutrients necessary for optimal development during pregnancy and early childhood (Pimpin et al., 2019). Due to the high bioavailability of some of these nutrients (Headey et al., 2018), ASFs are more effective in preventing stunting than other foods, but their production and consumption are associated with potential food safety risks (Li et al., 2019), linked to their perishability, chemical composition and microbiological status (Prache et al., 2022). Infection with *Salmonella* or other foodborne pathogens can lead to enteric infections, both asymptomatic and diarrheal, which are associated with stunting (Budge et al., 2019). Furthermore, enteric pathogens such as *Campylobacter*,* E. coli*,* Cryptosporidium* and *Giardia* have been linked to growth faltering and impact cognitive and metabolic development (Nataro & Guerrant, 2017). While ASF VCs can be integral to market-based solutions, they should not be promoted or scaled up without proper attention to eventual food safety and other risks.

Consequently, this study aimed to explore risks and opportunities in the egg VC with a view to alleviate stunting in Hyderabad, India. The egg VC was chosen because eggs, given their nutrient composition, have shown promising results to reduce stunting (Lutter et al., 2018). Moreover, eggs have become more affordable and available in the last twenty years, particularly in urban areas (Scudiero et al., 2023). They also constitute a source of foodborne illness through *Salmonella*, and other foodborne hazards (Chousalkar et al., 2021). In India, the large majority of reported bacterial foodborne disease outbreaks between 1980 and 2009 involved *Salmonella* (Vemula et al., 2012). The findings from this research contribute to the larger goal of mitigating stunting and its associated long-term consequences.

## Materials and methods

### Study area

The VCA was conducted in the low-income urban informal settlements of Addagutta and Warasiguda in Hyderabad, Telangana, India, which were selected as cohort study sites of the AASH programme (Davies-Kershaw et al., 2024) with cohort enrolment explained elsewhere (Kumar Banjara et al., 2024). The VC mapping started from the cohort sites with retailers the cohort mothers bought eggs from working backwards to egg producers (inside or outside of Hyderabad).

### Data collection

To identify the starting points for the VC mapping (i.e., retailers), six focus group discussions were conducted with a total of forty-eight pregnant or lactating mothers from cohort households in Addagutta and Warasiguda. The questions covered ASF consumption, financial and food safety aspects, quality and marketing, challenges faced, mapping of retailers and seasonality patterns. Details of the data collection and results are described elsewhere (Ravichandran, 2024). From these discussions, relevant key informants in the VC were identified, starting with local retailers. Subsequently, a snowballing approach was used to identify other relevant key informants and using the principle of sampling to saturation. Ten key informant interviews were conducted with two retailers, one trader, two wholesalers, three farmers, and two egg industry representatives.

The informants were interviewed from October 2022 to February 2023 using a question guide (supplementary material) enquiring about: (1) the physical location of markets, retailers, and roads in the community; (2) the origin, flows and destination of food and types of production, processing, and distribution channels; (3) product and marketing characteristics including differentiation attributes; (4) economic transactions and payment processes; (5) seasonality effects; (6) perceptions and behaviour of VC actors regarding food safety; and (7) rules and governance structures (informal and formal). It was tested with two key informants in a pilot study; adjustments in wording were made where the meaning was not completely clear. One interview was conducted in English and the others took place in Telugu and were transcribed in intelligent verbatim and translated into English by the interviewer.

### Conceptual framework development

**Fig. 1:**
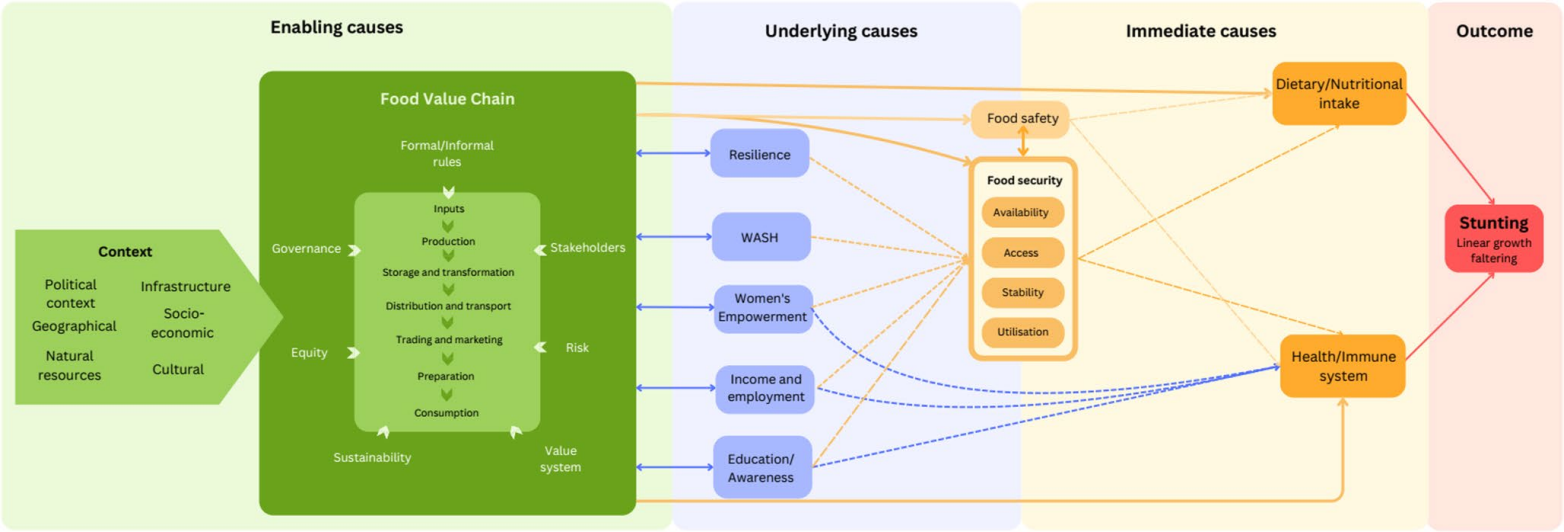
Conceptual framework: pathways linking value chains and stunting. Enabling causes include context and factors such as rules or value systems; they create the conditions for the food value chain (FVC) to operate. Underlying causes such as resilience (when compromised, it prevents stakeholders from responding adequately to stressors such as food insecurity) or water, sanitation and hygiene (WASH) conditions that contribute to stunting through sustained exposure to enteric pathogens are determined by the FVC, feed back into the FVC influencing its structure and feed forward into immediate causes. Immediate causes determine dietary intake and disease such as food security including its four pillars of availability, accessibility, stability and utilisation and food safety; they are mentioned by UNICEF (1993) as immediate causes of malnutrition. The pathways, although depicted separately, overlap and interact with each other

A conceptual framework (Fig. 1) was developed showing pathways linking VCs and stunting, as no such framework existed. This framework was used to analyse the egg VC from a nutrition-sensitive lens and to detect issues that could contribute to stunting.

The framework was created through an iterative and collaborative process between the authors based on a review of the existing literature. Relevant publications were obtained using combinations of relevant keywords on PubMed and Google Scholar, through snowballing from references included in the documents found and recommendations from the authors. Causes leading to stunting were classified from furthest to closest by following the pathways of connections and repercussions that ultimately lead to stunting; a brief explanation of the framework components is provided in the supplementary material.

### Data analysis

Reflexive thematic analysis (Braun & Clarke, 2021), was utilised to move data from a descriptive into an explanatory realm, generating themes to explain how VC activities could be linked to stunting. We took an ontological position of critical realism and an epistemological position of contextualism thereby recognising that access to the reality of stunting is impacted by both our interactions with interviewees and our theoretical positioning.

The six phases of thematic analysis were followed: familiarisation, coding, generation of initial themes, development and reviewing of themes, refining and naming themes, and writing up (Braun & Clarke, 2021). Data coding was conducted using the software QRS International NVivo (ver. 14.23.1). Analysis began deductively, using the lens of the conceptual framework followed by multiple rounds of coding and re-coding and theme generation around central organising concepts in an iterative process that took place between co-authors. The full details of the reflexive thematic analysis are provided in the supplementary material.

## Results

### Egg value chain structure

**Fig. 2:**
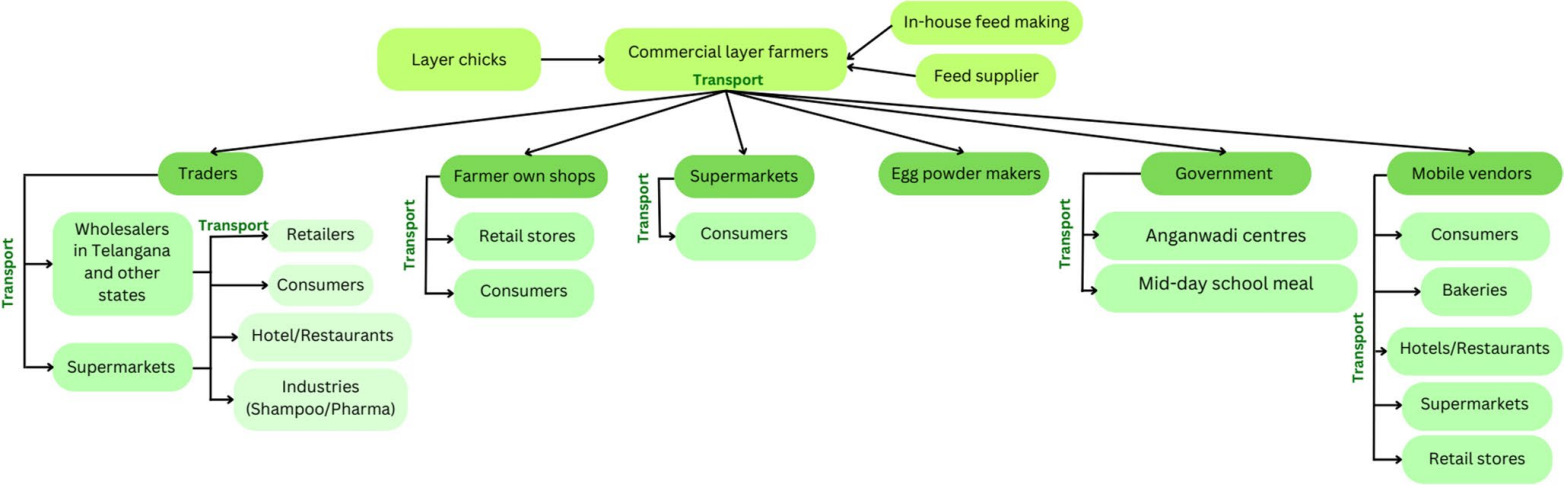
Overview of the egg VC structure in Hyderabad, India. Commercial layer farms sell eggs through their shops to consumers, traders, supermarkets, egg-powder makers, the government, and mobile vendors. The government distributes eggs to children and pregnant women via school meals and primary health centres known as Anganwadi centres (publicly funded childcare centres). Transporters operate at all stages of the VC. Traders and mobile vendors supply multiple stakeholders in the chain, in and out of Telangana state. Backyard egg production has been found to be negligible in this context

An overview of the egg VC structure in Hyderabad is provided (Fig. 2) to contextualise the results of the thematic analysis. A detailed description of the VC map can be found in Ravichandran (2024)

### Overview of themes

**Fig. 3:**
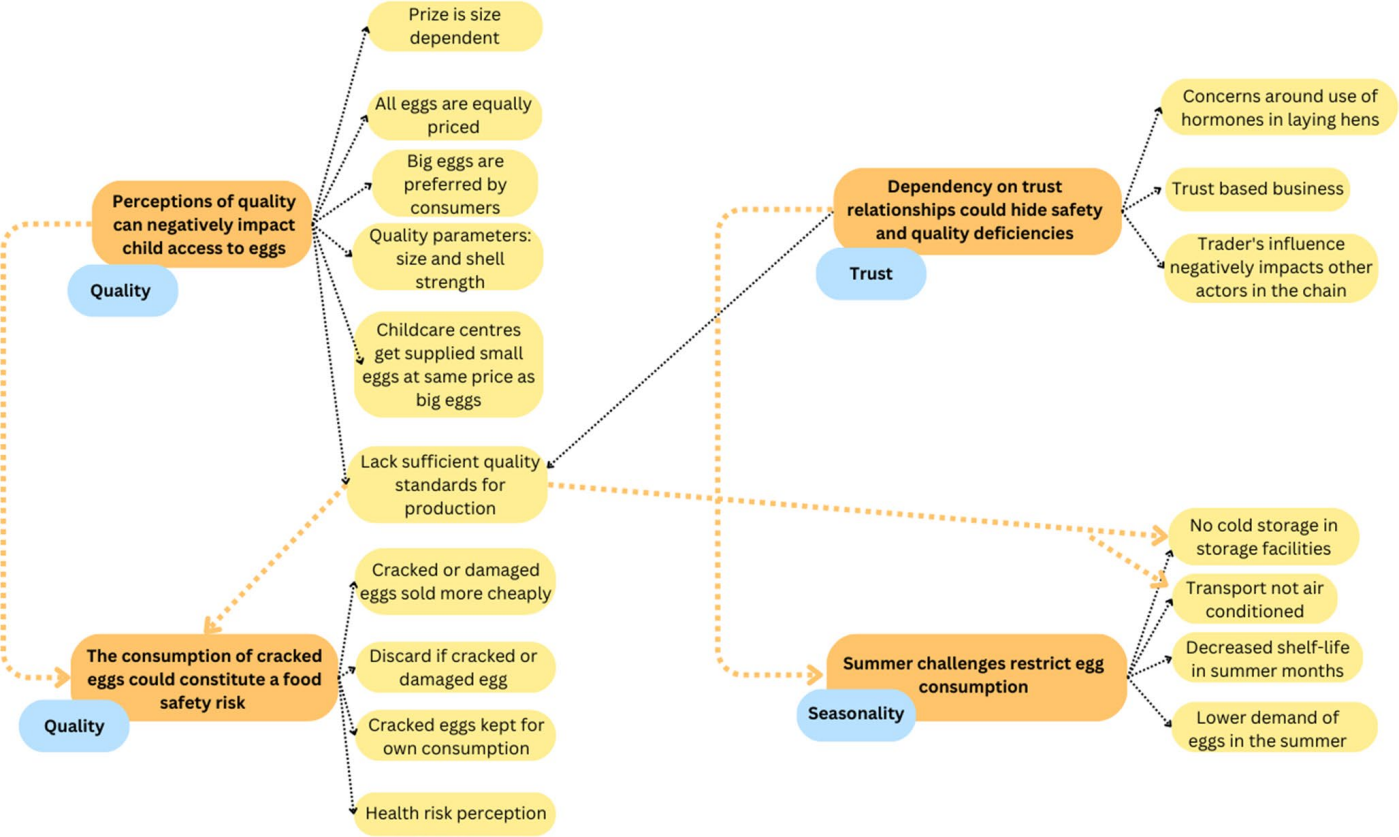
Overview of the four themes developed. The most commonly identified codes within the key informant interviews on the egg value chain, Hyderabad, India, are shown. Orange boxes: key themes; yellow boxes: examples of supporting codes for the themes; blue boxes: central organising concepts

Four themes were generated in relation to elements of the VC that could influence stunting (Fig. 3). The first two themes have quality as a central organising concept, focusing on perception of quality through physical characteristics and presence of damage; while the third and fourth themes have seasonality and trust, respectively, as their central organising concept.

#### Theme 1: perception of quality can negatively impact child access to eggs

This theme explores how VC stakeholders perceive egg quality, which is primarily through a visual assessment of size, and how this perception results in eggs moving differently through the VC.

Quality was perceived differently by different informants. In the mentioned absence of official quality standards, size and shell strength were considered the two main determinants of quality in eggs. These factors were described to influence consumer preferences, supply decisions by the stakeholders, and price.

Shell strength was considered by traders for transport purposes. Big eggs with weaker shells were transported for shorter distances as opposed to small eggs with stronger shells that could be transported further to different cities and states.

Concerning size, diverse messages around preferences and quality were delivered. Retailers reported that some consumers did not have preferences, whereas others looked for big eggs, as they associated large sizes with good quality. Despite egg size impacting demand, size was said not to affect purchase cost for consumers, but some farmers reported being paid a premium for bigger eggs when selling them to traders.

Other stakeholders held perceptions that eggs from backyard farming of local breeds were considered healthier and sold to consumers at a higher price than those from commercial breeds. Small eggs from local breeds were mentioned multiple times as being healthier, and that they should be given to children, but did not seem to be demanded by consumers purchasing eggs from retail shops. Farmers that have obtained a government contract to supply Anganwadi centres were described as selling to the government small eggs at the same price they sell big eggs to traders, making a higher profit:

*“[A] few farmers are cheating; they give small eggs to Anganwadi centres and charge full price. […] Traders buy small eggs from farmers saying that there is no demand and sell them at higher prices to the government. […] The fraud that is happening should be stopped as our children are affected.” Egg industry representative.*

These considerations around small eggs, transport, demand, and price could have an impact on the characteristics of eggs children have access to, and therefore on their nutrition.

#### Theme 2: consumption of cracked eggs could constitute a food safety risk

This theme explores how the VC deals with commodity damage. Instead of sub-standard eggs being discarded, a subsidiary chain emerges directed towards the poorer sections of society.

Egg deliveries were checked for damage or cracks by the buyers along the VC. Eggs arriving broken after transport were said to be replaced by the seller with undamaged eggs, but were not replaced if damaged after delivery, as they become the responsibility of the current owner. Because of the eggs’ fragility, a common practice described was to use trays and cotton to avoid breakage during transport and storage.

The broken eggs were said to be discarded, sold to food stalls or restaurants, or consumed in the household of the current owner of the eggs. When sold, they have a lower price than non-cracked eggs, creating a secondary market for broken eggs along the supply chain.

Some of the informants mentioned potential contamination of cracked eggs, specifically by *Salmonella*, and how they mitigate this risk by consuming the eggs on the day they crack and by boiling them, which seems to be common practice, avoiding potential negative impacts on health:

*“It’s not recommended to keep the cracked eggs for more than one day. There may be some chance [of] contamination*,* but we still boil the egg at maximum temperature before eating it so that it doesn’t affect our health.” Farmer two.*

The commodification of cracked eggs could constitute a food safety risk, as pathogens can enter the egg through the crack, potentially impacting children’s health and, by extension, stunting.

#### Theme 3: summer challenges restrict egg consumption

This theme explores how India’s egg VC is subject to seasonal variations that affect egg production and consumption. Egg production was described to decrease during the hot (summer) season due to heat stress affecting hens’ productivity with mitigation measures including coolers and dripping wet curtains somewhat decreasing mortality. Despite this drop in production, however, informants consistently stated that the abundance of farms in and around Hyderabad means that the supply of eggs can satisfy the demand during the hot season, which was said to be lower.

Informants mentioned a drop in egg demand during the hot season:

*“Due to cool weather*,* people prefer to eat more eggs [in the winter] compared to summer season” Retailer one.*

Another explanation for the diminished demand was egg spoilage with eggs said to last around one to two months in the cold season compared to one to two weeks in the hot one. An absence of air-conditioning or refrigeration practices, both during transport and storage, was described, leading to quick spoilage with the high temperatures reached during the hot season. Only supermarkets refrigerate eggs in the VC.

Spoiled eggs were reported to be discarded, representing a considerable loss for the retailers. In the egg business, farmers, wholesalers and retailers did not seem to consider air-conditioning during transport and cold storage as essential, and an informant deplored the absence of legislation or guidelines set by the government on this matter. Ventilation in shops and eggs and trays being clean were mentioned as part of good storage practices. Nevertheless, informants consistently explained that storage is not commonly used, as the business operates on an “in and out” basis, where farmers have limited storage capacity.

The short shelf-life of eggs and risk of spoilage due to the lack of a cold-chain in the egg VC during the hot season could impact the willingness of consumers to purchase eggs during this season, potentially depriving children of this nutritious food during this season and impacting, by extension, stunting.

#### Theme 4: dependency on trust relationships could hide safety and quality deficiencies

This theme explores how, in the absence of standards and guidelines, trust becomes a dominant feature of how the VC operates.

Most value chain informants mentioned a lack of standards and guidelines in the egg sector, especially in comparison to other countries. Changes and improvements in the egg sector quality are reportedly driven by consumer demands around the product, not by regulatory demands.

In the absence of standards and guidelines, business transactions seemed to depend on relationships and trust between stakeholders and their own judgement to run their business. Informants consistently described the creation of long-term relationships with other actors in the chain and trusting the quality of the product bought and the fairness of the price. Agreements between actors were almost exclusively verbal, with written contracts only existing between farmers and the government to supply Anganwadi centres.

However, issues of distrust were seen between some groups of stakeholders in the VC. Traders, because of their extensive influence in the business and their ability to set prices, were not seen in a good light by other stakeholders:

*“Farmers are forced to sell the egg at [a lower] price as asked or demanded by the traders. So*,* traders [take advantage of] the farmer’s weakness by asking […] [a lower] cost. Because farmers have no way to keep the stock with them.” Other official.*

Furthermore, it seems that the national poultry association has lost the trust of some informants, who perceived it to be controlled by other stakeholders. The government did not appear to be trusted either, with some informants mentioning instances of corruption. The lack of trust also related to farming practices, as some stakeholders believed hormones were used on laying hens and that this could impact consumers’ health. When these concerns were raised during the interviews, they were dismissed by farmers and their associations as myths created by marketing companies.

There was a perception that the government knew little about what was happening in farms as visits by government officials were infrequent. Informants denied rumours of hormones being used or the presence of aflatoxins and antibiotic residues, yet there appeared to be no official inspections to verify these claims.

Inspections were said to be conducted at the retailer’s level only and to be very superficial. The lack of sufficient inspections in the different steps of the VC could mean that the quality and safety of eggs, as well as the production practices, cannot be ascertained.

## Discussion

Thematic analysis of interviews within the food VC stunting framework generated four key themes around the core concepts of quality, seasonality, and trust in the egg VC.

Consumer preferences influence egg demand, favouring larger eggs, despite some stakeholders mentioning the health benefits of smaller ones. Traders and farmers manage to get around the lower demand for small eggs by selling them to the Anganwadi centres at a good price.

Anganwadi centres provide eggs to children from the most disadvantaged families (NIPCCD, 2018), so the eggs’ quantity of nutrients and microbiological contamination could potentially impact children’s overall daily nutrient intake. Nevertheless, previous research did not show clear evidence of differences in terms of nutritional impact between consuming small and big eggs, from older or younger hens (Réhault-Godbert et al., 2019; Travel et al., 2011) and found mixed results on microbiological contamination of eggs in relation to the age of the hen (Kretzschmar-McCluskey et al., 2008; Huneau-Salaün et al., 2010; Vlčková et al., 2018) indicating that these factors may not affect children negatively. Moreover, eggs are boiled before being given to children in Angawadi centres, which can eliminate contaminating microorganisms (EFSA, 2014).

Eggs with cracks on their shells were found to be commercialised and consumed. Consumption of cracked eggs is reported to happen at the level of farmers and retailers. Farmers and retailers consume these eggs themselves or give them to their workers or sell them to street food vendors at a lower price. Cracked eggs may be consumed by children through these three paths. While informants noted *Salmonella* could contaminate eggs, they reported always boiling them to prevent any health problems. Some studies found a prevalence of 7.7% (Suresh et al., 2006) and 4.82% (Singh et al., 2010) of *Salmonella* in raw eggs in India, with *Salmonella* Enteritidis being the predominant serovar found. There is limited literature on egg-boiling practices and the consumption of cracked eggs in India or other countries. An article reported that in Nigeria cracked eggs are sold at about half the price of whole uncracked eggs, and cracks were found to increase significantly the load of bacterial groups and species present (Edema & Atayese, 2006). During preparation, storage and handling there is potential for cross-contamination of surfaces and other foods (Cardoso et al., 2021). The consumption and manipulation of cracked eggs could therefore present a health risk for children, either through inadequate cooking or cross-contamination of surfaces and foods.

Consumption of eggs decreases during summer due to reduced productivity and lowered demand, as consumers perceive hens to be at a higher risk of disease during this season (Scudiero et al., 2023). The drop in demand outweighs the supply reduction and leads to lower prices in those months (Karthikeyan & Nedunchezhian, 2014). Indian consumers consider eggs as food that produces more body heat during digestion (Karthikeyan & Nedunchezhian, 2014), which is considered impure by Upper Caste Hindu groups (Scudiero et al., 2023). Egg allergy was mentioned as a possible explanation for increased body heat, but this has not been associated with hot weather in the literature. The prevalence of allergies in Asian individuals is similar to or lower than that of Western countries (Lee et al., 2013; Hossny, 2019).

Egg spoilage also increases in the hot season due to the lack of a cold chain and high temperatures. Previous studies identified the lack of a cold chain in storage and transport as a weakness in the Indian egg supply chain (Mitra et al., 2021; DAHDF, 2017), with increased spoilage during the hot season being mentioned by Travasso et al. (2023). The absence of a cold chain favours microbial growth on the surface of the eggshell, which can cause cross-contamination of surfaces and foods, and even penetration of eggshell and contamination of the internal contents (Chousalkar et al., 2021). Additionally, a lack of refrigeration impacts egg quality, reduces egg weight and affects yolk weight and pH (Akter et al., 2014).

If not replaced by other nutritious food, a decrease in egg consumption could lead to a reduced nutritional intake during these months, potentially impacting stunting. Additionally, the potentially reduced egg quality and food safety (WHO, 2017) could be a cause of stunting, as these eggs are being exposed to higher temperatures. In any case, concerns around heat and allergies may warrant further investigation, as pushing for higher egg consumption could in turn create other health concerns.

Trust relationships between actors appeared to be filling the gap left by the lack of official standards and government regulations in the egg VC. The lack of sufficient standards, or enforcement of those existing, has been reported previously by Mitra et al. (2021). The absence of quality standards and legislation regarding practices along the chain could negatively impact food safety, favouring stunting (Li et al., 2019). In India, neither the government nor the self-regulating industry bodies have established quality standards for farm management. The national action plan for the sector (DAHDF, 2017), while stating egg production goals and planning to scale up backyard farming into larger scale poultry farming in the next years, gives little indication on how to achieve this, and mainly mentions improvements in standards related to egg exports. Concerns about hormone or antibiotic use and the presence of aflatoxins in feed were critical points of discussion and have been highlighted in previous studies of poultry production in India (Walia et al., 2019; Kannan et al., 2014). Our findings are consistent with a previous study by Deena and Sivanesan (2019) who reported distrust in Anganwadi centre workers and the government by workers themselves due to instances of corruption. The study by De Vries et al. (2023) on trust in agricultural VC mentions how power inequalities among VC actors and a lack of a transparent information flow can lead to distrust, which corresponds to what is happening in Hyderabad’s egg VC in relation to food safety. Attention to trust is relevant if market-based interventions are to increase the (safe) consumption of eggs, particularly by children.

In the absence of published frameworks linking VCs and stunting, a new model was developed to guide this analysis. It offers a structured, context-specific approach to understanding stunting’s complexities and identifying opportunities and barriers for intervention (De la Peña & Garrett, 2018) and provides a holistic view of VC–stunting links in the target community (Allen et al., 2016). Although its single-commodity focus is a limitation, the framework allows exploring a wide range of VC-related factors that influence child stunting and can therefore yield valuable insights into ASFs, which are being increasingly promoted for their nutritional benefits.

The findings were included in a stakeholder workshop discussing and validating the wider VC findings and suggesting potential entry points for improvement of the egg and dairy VCs in Hyderabad. The identified VC practices, ranging from consumer preferences and seasonal fluctuations to handling of cracked eggs and trust dynamics, highlight multiple pathways through which the egg VC may influence child stunting. These practices affect both the nutritional quality and safety of eggs, which may reduce nutrient intake or increase exposure to foodborne pathogens for children. While direct causality cannot be established within this qualitative framework, the findings show plausible mechanisms linking VC practices to stunting risks that can be investigated further. In this case, they were used to inform the development of a sampling frame to test eggs in multiple points of the VC for contamination with foodborne pathogens and chemical hazards. The insights from the stakeholder workshop and biological sampling and testing will be reported separately. Details of dietary practices, stunting, and food safety and WASH risk factors in the home environment collected in the AASH cohort study will become available in the future and can also be linked to the results of this study.

## Conclusion

Our egg value chain analysis in Hyderabad reveals several critical issues potentially linking value chain activities with stunting. The widespread absence of standards and regulations and the trust-based system highlight a challenging environment for food safety. Stakeholders’ individual perceptions drive business decisions and influence demand, contributing to egg quality variations. Limited knowledge of egg safety, regarding chemical residues, toxins, and microbiological contamination (particularly in cracked eggs) highlights areas where improvements may be needed. Infrastructure gaps, such as inadequate storage and cold chains, affect farmers’ bargaining power and egg quality, especially in summer. Applying a nutrition-sensitive lens to our value chain analysis allowed the identification of opportunities and barriers related to stunting, highlighting the potential of such an approach in creating a holistic understanding of stunting causes and identifying solutions.

## Supplementary information

Below is the link to the electronic supplementary material.

ESM 1 (DOCX 4.31 MB)
