# Supplementary material for: Hyderabad’s egg value chain: investigating potential influences on childhood stunting
Source: Food Secur. 2026 Jan 31;18(2):483–94. doi: 10.1007/s12571-025-01633-6 (PMC13031228; doi:10.1007/s12571-025-01633-6)
Supplement: Supplementary file 1 — (DOCX 4.31 MB) [file 12571_2025_1633_MOESM1_ESM.docx]

**SUPPLEMENTARY MATERIAL**

**Journal: Food Security**

**Hyderabad's egg value chain: investigating potential influences on childhood stunting**

**Emma Gomez de Gracia^1^, Barbara Häsler^1^, Srinu Rotta^2^, Archana Konapur^2^, Thanammal Ravichandran^3^, Paula Dominguez-Salas^4,5^, Mathew Hennessey^1^, Delia Randolph^4,5^, Little Flower Augustine^2^, Naveen Kumar Ramachandrappa^2^, Claire Heffernan^6^, Santosh Kumar Banjara^2^, Bharati Kulkarni^2^**

**Affiliations**

^1^ Veterinary Epidemiology Economics and Public Health Group, Department of Pathobiology and Population Sciences, Royal Veterinary College, London, United Kingdom

^2^ ICMR-National Institute of Nutrition, DHR, Ministry of Health and Family Welfare, GoI, Tarnaka, Beside Tarnaka metro station, Hyderabad, Telangana, India

^3^ Department of Social Works, Kumaraguru College of Liberal Arts and Science, Coimbatore, Tamil Nadu, India

^4^ Natural Resources Institute, University of Greenwich, London, UK

^5^ International Livestock Research Institute, Nairobi, Kenya

^6^ London International Development Centre, London School of Hygiene and Tropical Medicine, London, UK

**Corresponding author**:

* Barbara Häsler. E-mail address: [bhaesler@rvc.ac.uk](mailto:bhaesler@rvc.ac.uk)

**Section 1**

*Brief explanation of the conceptual framework components*

| Type of cause | Causes | Description |
| --- | --- | --- |
| Enabling causes | Context | Geographical, political, cultural, and socio-economic context, as well as the infrastructure and natural resources specific to a time and place (Nicholson et al., 2020). |
|  | Value chain (VC) | The VC of a commodity includes the steps from the inputs needed to produce it to its consumption, reflecting the creation and distribution of value along it (Ruel et al., 2013). |
|  | Factors involved in the VC | Value can be economic or refer to the nutritional value of a product, depending on which lens we are using to analyse the chain (De la Peña & Garret, 2018). |
|  |  | Governance refers to the power relations between the different stakeholders (Kaplinsky & Morris, 2001). |
|  |  | Formal rules refer to legislative requirements and informal rules refer to rules promoted by standards expected from key actors of the chain (Kaplinsky & Morris, 2001). |
|  |  | Equity, while desired, is not commonly found, making looking for inequities a useful aspect of the VC analysis (Gillespie & van den Bold, 2017). |
|  |  | Reducing vulnerability and risk for chain participants is important to incentivise participation (Maestre Morales & Poole, 2018). |
|  |  | Sustainability is achieved when a VC is profitable over the longer term while not permanently depleting natural resources, being adaptable to climate and socio-economic changes (Allen et al., 2016). |
| Underlying causes | Resilience | The capacity to adequately respond to shocks and stressors. When compromised, it prevents stakeholders from responding to climatic shocks or stressors such as corruption or insecurity (Béné, 2020). |
|  | Water, sanitation and hygiene (WASH) | WASH conditions contribute to stunting through sustained exposure to enteric pathogens and through socio-economic pathways, including the diversion of household income from food budgets (Cumming & Cairncross, 2016). |
|  | Women’s empowerment | Women allocate most of their household money to health and nutrition for their children (Raza et al., 2020). They influence child stunting through their own nutrition and health during pregnancy and breastfeeding (Henson & Humphrey, 2015), the determinants of their malnutrition overlapping with those of children (UNICEF, 2021). |
|  | Employment and income | Employment and increased income can be translated into expenditure on nutrient-dense, diversified food, healthcare and education (Gillespie & Van den Bold, 2017). |
|  | (Nutritional) Education and Awareness | Higher levels of education increase the possibility of employment (Gelli et al., 2015), while nutritional education favours practices that promote food safety and demand for nutrient-dense products (Gelli et al., 2015). |
| Underlying- Immediate causes | Food security | “Food security exists when all people, at all times, have physical and economic access to sufficient, safe and nutritious food that meets their dietary needs and food preferences for an active and healthy life” (FAO, 2006). These elements are explained in the following rows. |
|  | Four pillars of food security | Availability “of sufficient quantities of food of appropriate quality” (FAO, 2006), referring also to the availability of food year-round, within physical time and distance (El Bilali et al., 2019). |
|  |  | Accessibility is the “access by individuals to adequate resources (…) for acquiring appropriate foods for a nutritious diet (…)” (FAO, 2006). Nutritious food not only needs to be available but also economically accessible (Ridoutt et al., 2019). |
|  |  | Stability implies that “a population, household or individual must have access to adequate food at all times (…)” (FAO, 2006). It is affected by periods of economic constraint (Gelli et al., 2019) and by foodborne pathogens that can have cyclical or seasonal patterns (Häsler et al., 2017). |
|  |  | Utilisation “of food through adequate diet, clean water, sanitation and health care to reach a state of nutritional well-being where all physiological needs are met” (FAO, 2006). This is dependent on the convenience and desirability of a product to the consumers (De la Peña & Garret, 2018), as well as on nutritional quality (Häsler et al., 2017). |
|  | Food safety | Impacts all pillars of food security (Häsler et al., 2017) and refers to the absence of contaminants in the food such as bacteria, viruses, mycotoxins or chemicals (Grace, 2015). Foodborne pathogens can inhibit nutrient absorption, debilitating the immune system and worsening infections (Budge et al., 2019). |
| Immediate causes | Dietary/nutritional intake | Includes nutrient and caloric content, dietary diversity, and presence of anti-nutrients (WHO, 2017). |
|  | Health and immune system | Includes diarrheal disease, environmental enteropathy dysfunction and respiratory infection among others (WHO, 2017). |
| Outcome | Stunting | Measured as linear growth faltering. |

**References**

Allen, S. L., de Brauw, A. & Gelli, A. (2016). Nutrition and sustainability: Harnessing value chains to improve food systems. In: 2016 Global Food Policy Report, chapter 6, 48-55, International Food Policy Research Institute (IFPRI), Washington, D.C. <https://doi.org/10.2499/9780896295827>

Béné, C. (2020). Resilience of local food systems and links to food security - A review of some important concepts in the context of COVID-19 and other shocks. *Food Secur.*, *12*(4), 805–822. <https://doi.org/10.1007/s12571-020-01076-1>

Budge, S., Parker, A. H., Hutchings, P. T. & Garbutt, C. (2019). Environmental enteric dysfunction and child stunting. *Nutr. Rev.*, *77*(4), 240–253. <https://doi.org/10.1093/nutrit/nuy068>

Cumming, O. & Cairncross, S. (2016). Can water, sanitation and hygiene help eliminate stunting? Current evidence and policy implications. *Matern Child Nutr.*, *12* (Suppl 1), 1-105. <https://doi.org/10.1111/mcn.12258>

De la Peña, I. & Garrett, J. (2018). Nutrition-sensitive value chains. A guide for project design. Volume I, IFAD. Retrieved March 10, 2025, from <https://www.ifad.org/en/web/knowledge/-/publication/nutrition-sensitive-value-chains-a-guide-for-project-design>

El Bilali, H., Callenius, C., Strassner, C. & Probst, L. (2019). Food and nutrition security and sustainability transitions in food systems. *Food Energy Secur.*, *8*(2), e00154. <https://doi.org/10.1002/fes3.154>

FAO (Agriculture and Development Economics Division, ESA) (2006). *Policy Brief on Food Security.* Retrieved March 10, 2025, from <https://www.fao.org/fileadmin/templates/faoitaly/documents/pdf/pdf_Food_Security_Cocept_Note.pdf>

Gelli, A., Donovan, J., Margolies, A., Aberman, N., Santacroce, M., Chirwa, E., Henson, S. & Hawkes, C. (2019). Value chains to improve diets: Diagnostics to support intervention design in Malawi. *Glob. Food Secur.*, *25*, 100321. <https://doi.org/10.1016/j.gfs.2019.09.006>

Gelli, A., Hawkes, C., Donovan, J., Harris, J., Allen, S., de Brauw, A., Henson, S., Johnson, N., Garrett, J. & Ryckembusch, D. (2015). Value Chains and Nutrition. A Framework to Support the Identification, Design, and Evaluation of Interventions, IFPRI Discussion Paper 1413. International Food Policy Research Institute (IFPRI), Washington, D.C. [http://dx.doi.org/10.2139/ssrn.2564541](https://dx.doi.org/10.2139/ssrn.2564541)

Gillespie, S. & van den Bold, M. (2017). Agriculture, Food Systems, and Nutrition: Meeting the Challenge. *Global Chall.*, *1*(3), 1600002. <https://doi.org/10.1002/gch2.201600002>

Grace, D. (2015). Food Safety in Low and Middle Income Countries. *Int. J. Environ. Res. Public Health*, 12(9), 10490–10507. <https://doi.org/10.3390/ijerph120910490>

Häsler, B., Dominguez-Salas, P., Fornace, K., Garza, M., Grace, D. & Rushton, J. (2017). Where food safety meets nutrition outcomes in livestock and fish value chains: a conceptual approach. *Food Secur.*, *9*, 1001–1017. <https://doi.org/10.1007/s12571-017-0710-2>

Henson, S. & Humphrey, J. (2015). Review of Agri-Food Value Chain Interventions, LANSA Working Paper Series, 2015(4). Retrieved March 10, 2025, from <https://assets.publishing.service.gov.uk/media/57a0898de5274a27b200012f/LANSA_WP4_December_2015_final.pdf>

Kaplinsky, R. & Morris, M. (2001). A Handbook for Value Chain Research. Institute of Development Studies, University of Sussex, Brighton, UK. Retrieved March 10, 2025, from <https://www.fao.org/fileadmin/user_upload/fisheries/docs/Value_Chain_Handbool.pdf>

Nicholson, C. F., Kopainsky, B., Stephens, E. C., Parsons, D., Jones, A. D., Garrett, J. & Phillips, E. L. (2020). Conceptual frameworks linking agriculture and food security. *Nat. Food*, *1*(9), 541–551. <https://doi.org/10.1038/s43016-020-00142-3>

Maestre Morales, M. & Poole, N. (2018). Introduction: Value Chains for Nutrition in South Asia: Who Delivers, How, and To Whom? *IDS Bulletin*, *49*(1), 1-20. <https://doi.org/10.19088/1968-2018.100>

Raza, A., Fox, E. L., Morris, S. S., Kupka, R., Timmer, A., Dalmiya, N. & Fanzo, J. (2020). Conceptual framework of food systems for children and adolescents. *Glob. Food Secur.*, *27*, 100436. <https://doi.org/10.1016/j.gfs.2020.100436>

Ridoutt, B., Bogard, J. R., Dizyee, K., Lim-Camacho, L. & Kumar, S. (2019). Value Chains and Diet Quality: A Review of Impact Pathways and Intervention Strategies. *Agriculture*, *9*(9), 185. <http://dx.doi.org/10.3390/agriculture9090185>

Ruel, M. T., Alderman, H. & The Maternal and Child Nutrition Study Group (2013). Nutrition-sensitive interventions and programmes: how can they help to accelerate progress in improving maternal and child nutrition? *Lancet*, *382*(9891), 536–551. <https://doi.org/10.1016/S0140-6736(13)60843-0>

UNICEF (2021). *UNICEF Conceptual Framework on Maternal and Child Nutrition*. Retrieved March 10, 2025, from <https://www.unicef.org/media/113291/file/UNICEF%20Conceptual%20Framework.pdf>

WHO (World Health Organization) (2017). Stunted Growth and Development: The Conceptual Framework – Childhood Stunting: Context, Causes and Consequences. Retrieved March 10, 2025, from <https://www.who.int/publications/m/item/childhood-stunting-context-causes-and-consequences-framework>

**Section 2**

*Application of a reflexive thematic analysis (TA) to the processing of the qualitative data obtained from the interviews.*

Reflexive thematic analysis (TA) was used to process the qualitative data obtained from the interviews, as described by Braun and Clarke (2021). It aligns with Big Q qualitative work (Alvesson and Sköldberg, 2009), utilising qualitative techniques for data generation informed by qualitative research values (Braun and Clarke, 2023). Reflexive TA was chosen because of its theoretical flexibility, meaning that it could encompass a wide range of theoretical positionings. It also allowed for flexibility in the coding process since, not being restricted by a pre-existing codebook, it could be modified as researchers were engaging and working through the data. Furthermore, it focuses on creating contextualised and situated knowledge aimed at getting an understanding of the local situation (Braun and Clarke 2021).

The analysis was developed deductively through the lens of the conceptual framework presented, the qualitative framework being experiential, as the objective was to explore individuals’ perspectives and understanding of the topic. As for the theoretical framework, the ontological positioning was critical realist and the epistemological positioning was contextualism (Braun and Clarke 2021).

Familiarisation was achieved through repetitively reading the transcripts and creating maps for each interview on paper to get fully immersed in the data. Data coding was done with QRS International NVivo (ver. 14.23.1 (38)) software, through multiple rounds of coding and re-coding, discussed in regular meetings, to allow for discussion and reflection on the data at hand. A mixture of semantic and latent coding was obtained, working systematically through the interviews to identify data segments potentially relevant to the research questions. Theme generation and reviewing were done firstly by creating summary themes, allowing for the data to be organised around the main topics discussed. From there, through conversations and drawing across summary themes, codes, and transcripts, candidate themes were developed identifying patterns of meaning. In order to obtain the final themes, during a visit to Hyderabad candidate themes were presented to members of the research team so that they could be validated, discussed and refined taking advantage of their extensive knowledge of the context. A reflexivity journal was kept throughout the research project to reflect on its different aspects and to consider and interrogate the researchers’ own way of thinking and positionality in relation to the project. The main areas of focus were personal, interpersonal, methodological and contextual as guided by Olmos-Vega *et al.* (2023). Personal reflexivity is focused on the researcher’s assumptions about context, participants or data. It considers how researchers are affecting the research and vice versa. Interpersonal reflexivity refers to how the relationships between researchers and participants influence the results. Methodological reflexivity focuses on considering the methodological choices of the authors and their repercussions on the project. The last type is contextual reflexivity, which refers to situating the work in its cultural and historical context (Olmos-Vega *et al.* 2023).

**References**

Alvesson, M. & Sköldberg, K. (2009). Reflexive Methodology: New Vistas for Qualitative Research, 2^nd^ ed. SAGE Publications Ltd, London.

Braun, V., Clarke, V., 2021. Thematic Analysis: A Practical Guide, SAGE Publications Ltd, London.

Olmos-Vega, F. M., Stalmeijer, R. E., Varpio, L. & Kahlke, R. (2023). A practical guide to reflexivity in qualitative research: AMEE Guide No. 149, *Medical Teacher*, *45*(3), 241-251, <https://doi.org/10.1080/0142159X.2022.2057287>

**Section 3**

**Key informant interviews**

Instructions for interviewers are presented in *italic*

**Activity: Value chain mapping with all actors of the value chain (egg and milk)**

*This activity should be conducted with a key informant who has an overall understanding of the selected ASF production and distribution system. This could be a representative of association of different stakeholders such as vendors, farmers etc. Some of the stakeholders will have knowledge of part of the value chain only. The aim of these interviews is to get clear understanding of the egg and milk value chain in India especially area in which selected cohorts are living.*

*The interviewer will present a preliminary map of the value chain obtained from literature review*

- *This is an interactive process and both interviewer and the key informant need to agree on what should be written/added in the diagram.*
- *The starting point of the diagram can be variable and depends on the expertise of the key informant. Hence, if we are interviewing a vendor, then the starting point can be the type of vendors and who supply them and the products and follow from there. The map continues till we got all information the key informant is aware of.*

*The aim here is to have a diagram that shows the flow of selected ASF according to the: 1) type of actors involved, 2) type of nodes or places, 3) type of products and 4) temporal patterns.*

We would like you to help us draw a diagram that shows how *[selected ASFs]* that consumers living in Addagutta/other location access are produced and distributed in the region.

Please describe what kind of actors are involved in producing and distributing the *[selected ASFs]* and the links between them. *(Each network will have a specific type of actors operating, and these may get their supply or distribute products in a different way.)*

For each actor identified/you know, could you indicate if there are different types or groups based on: (Interviewer can ask all these details based on knowledge of the actor and his network)

- - Operation/business size or business type (For example: There might be different types of *selected ASF* traders: those operating with large quantities, those associated to a major group or association, those independent, etc.)
  - Knowledge and expertise
  - Seasonality
  - Geographical location (e.g. those who are local, those that operate at national level, those who specialise in wholesale from markets)
  - Value addition activity (e.g. labelling, cleaning, processing) *for dairy sector focus only on liquid milk i.e., raw, boiled and pasteurised milk*
  - Infrastructure (e.g. water source and access, dumping sites, fixed or mobile premises)
  - Technology used
  - Type of organization (companies, independent, etc.)
  - Socio-economic status of producers (e.g. income, ethnicity, gender, caste, landlessness, etc.)
    - Type of breeder (only for eggs)
  - Type of feed supplier
  - Type of market
  - Type of processor
  - Wholesale trader
  - Type of retailer
  - Type of consumer

*Once the diagram has been discussed and agreed upon (existing value chain map can also be used), ask the following questions:*

Please indicate what proportion of the flow goes through each arrow (e.g. 30% of eggs/milk from certain type of production system goes to a poultry market or is industrially packed). If proportion is not possible, please indicate which routes have highest, medium, and lowest amount of [selected ASFs] moving along.

Please describe seasonal variations of the flow *(prompts – rain, temperature and religious festivals and other cultural occasions).*

**Semi-structured questionnaire for vendors (hawkers/ small shops/ milk vendors)**

*It is recommended that the interview is structured in relation to one ASF. If other ASFs come up during the conversation, it is fine to let people talk about these as well and bring out interesting points (e.g., comparisons, good / bad practice examples, etc.), but then try to bring them gently back to the ASF chosen.*

*ఒక ASFకి సంబంధించి ఇంటర్వ్యూ నిర్మాణాత్మకంగా ఉండాలని సిఫార్సు చేయబడింది. సంభాషణ సమయంలో ఇతర ASFలు వచ్చినట్లయితే, ప్రజలు కూడా వీటి గురించి మాట్లాడటానికి అనుమతించడం మరియు ఆసక్తికరమైన అంశాలను (ఉదా., పోలికలు, మంచి / చెడు అభ్యాస ఉదాహరణలు మొదలైనవి) బయటకు తీసుకురావడం మంచిది, కానీ వాటిని సున్నితంగా తిరిగి మొదట ఎంచుకున్న ASFకి తీసుకురావడానికి ప్రయత్నించండి.*

*Selected ASF: Eggs or milk - ask only about one ASF to vendor even if they sell both ASF.*

General - సాధారణ సమాచారం

1. Who are the major suppliers for [selected ASF] to the vendors in this area and where are they based (prompts: district and state)? Can you describe these vendors? Prompts: size, influence, structure

ఈ ప్రాంతంలోని విక్రేతలకు గుడ్డు/పాలు ప్రధాన సరఫరాదారులు ఎవరు మరియు వారు ఎక్కడ ఉన్నారు (ప్రాంప్ట్‌లు: జిల్లా మరియు రాష్ట్రం)? మీరు ఈ విక్రేతల గురించి వివరించగలరా? ప్రాంప్ట్‌లు: పరిమాణం, ప్రభావం, నిర్మాణం

1. Do most of the vendors know the origin of the stock they receive (e.g.: type of farm, basic information about the farm)

చాలా మంది విక్రేతలకు వారు అందుకున్న స్టాక్ మూలం గురించి తెలుసా (ఉదా: పొలం రకం, పొలం గురించి ప్రాథమిక సమాచారం)?

1. Who are the major stakeholders, other than direct suppliers involved in providing you / vendors with [selected ASF]? And what are their roles? *(Can refer back to the diagram)*

మీకు / విక్రేతలకు [గుడ్డు/పాలు] అందించడంలో ప్రధాన సరఫరాదారులు కాకుండా ఇతర ప్రధాన లబ్దిదారుల ఎవరు? మరియు వారి పాత్రలు ఏమిటి? (రేఖాచిత్రాన్ని తిరిగి చూడవచ్చు)

Quality of ASF

1. How do vendors evaluate the quality of [selected ASF]?

విక్రేతలు [గుడ్డు/పాలు] నాణ్యతను ఎలా అంచనా వేస్తారు?

1. *Dairy sector India only:* There are frequently news reports about milk sold in the country being adulterated. It is said nearly 75% of the milk is adulterated. What do you think about the issue? Where do you think adulteration happens? Where geographically and where in the chain? How do you think they do it? What are the penalties if a vendor is found to sell adulterated milk? Do you think those penalties are enough to discourage adulteration?

దేశంలో విక్రయించే పాలు కల్తీ అవుతున్నాయని తరచూ వార్తలు వస్తున్నాయి. దాదాపు 75% పాలలో కల్తీ ఉందని చెబుతున్నారు. ఈ సమస్య గురించి మీరు ఏమనుకుంటున్నారు? కల్తీ ఎక్కడ జరుగుతుందని మీరు అనుకుంటున్నారు? భౌగోళికంగా ఎక్కడ మరియు గొలుసులో ఎక్కడ? వారు దీన్ని ఎలా చేస్తారని మీరు అనుకుంటున్నారు? విక్రయదారుడు కల్తీ పాలను విక్రయించినట్లు తేలితే జరిమానాలు ఏమిటి? కల్తీని అరికట్టడానికి ఆ జరిమానాలు సరిపోతాయని మీరు అనుకుంటున్నారా?

1. Is there a difference in quality between different suppliers?

వివిధ సరఫరాదారుల మధ్య నాణ్యతలో తేడా ఉందా?

- - If yes, what are the differences? Why do you think such differences exist?

అవును అయితే, తేడాలు ఏమిటి? అలాంటి తేడాలు ఎందుకు ఉన్నాయని మీరు అనుకుంటున్నారు?

- - What do vendors generally do with poor quality [selected ASF] (prompts: return to supplier, discard, or sell for less price or target different consumers?)

అమ్మకందారులు సాధారణంగా నాణ్యత లేని [గుడ్డు/పాలు]తో ఏమి చేస్తారు (ప్రాంప్ట్‌లు: సరఫరాదారుకి తిరిగి ఇవ్వడం, పారవేయడం లేదా తక్కువ ధరకు విక్రయించడం లేదా వివిధ వినియోగదారులను లక్ష్యంగా చేసుకోవడం??)

1. What is a good / bad quality [selected ASF]? What would be the price for a good one and for a bad one?

మంచి/చెడు నాణ్యత [పాలు/గుడ్డు] అంటే ఏమిటి? మంచిదానికి, చెడ్డవాటికి ఎంత ధర ఉంటుంది?

1. Are there certification schemes that ensure product quality?

ఉత్పత్తి నాణ్యతను నిర్ధారించే ధృవీకరణ పథకాలు ఉన్నాయా?

If yes, how available are these for the vendors in Addagutta and similar areas?

How frequently are they sought by vendors?

అవును అయితే, అడ్డగుట్ట/ ఇలాంటి ప్రాంతాల్లోని విక్రేతలకు ఇవి ఎంతవరకు అందుబాటులో ఉన్నాయి? వారు ఎంత తరచుగా విక్రేతలు కోరుతున్నారు??

1. In the diagram, do products with different quality go through different channels?

రేఖాచిత్రంలో విభిన్న నాణ్యత కలిగిన ఉత్పత్తులు వివిధ మార్గాల ద్వారా వెళ్తాయా?

- - If yes, why do you think that is? What differentiates actors involved in trading good quality [Selected ASF] from actors involved in trading poor quality [Selected ASF]? (Prompts: operation/business size, knowledge and expertise, geographical location, infrastructure and technology used, socio-economic status - income, ethnicity, gender, caste, landlessness, etc.)

అవును అయితే, అది ఎందుకు అని మీరు అనుకుంటున్నారు? నాణ్యమైన [పాలు/గుడ్డు] వ్యాపారంలో పాల్గొనే వ్యక్తులు మరియు నాణ్యత లేని [పాలు/గుడ్డు] వ్యాపారం చేసే వ్యక్తులు మధ్య తేడా ఏమిటి? (ప్రాంప్ట్‌లు: ఆపరేషన్/వ్యాపార పరిమాణం, అవగాహన మరియు నైపుణ్యం, భౌగోళిక స్థానం, ఉపయోగించిన మౌలిక సదుపాయాలు మరియు సాంకేతికత, సామాజిక-ఆర్థిక స్థితి - ఆదాయం, జాతి, లింగం, కులం, భూమిలేనితనం మొదలైనవి)

- - Can you describe these routes in the diagram?

మీరు ఈ మార్గాలను రేఖాచిత్రంలో వివరించగలరా?

Availability and seasonality

1. Please describe the effect of seasonality on availability of [selected ASF]?

దయచేసి [పాలు/గుడ్డు] లభ్యతపై కాలానుగుణ (వివిధ కాలాలలో) ప్రభావాన్ని వివరించండి?

- - Why do you think that is?

అది ఎందుకు అని మీరు అనుకుంటున్నారు?

- - How do you think vendors cope with it? Is there difference in coping mechanisms between largescale and small-scale vendors?

విక్రేతలు దీన్ని ఎలా ఎదుర్కొంటారని మీరు అనుకుంటున్నారు? పెద్ద స్థాయి మరియు చిన్న-స్థాయి విక్రేతల మధ్య కోపింగ్ మెకానిజమ్స్‌లో తేడా ఉందా?

1. Please describe the seasonality of price of [selected ASF]?

పాలు/గుడ్డు యొక్క ధర కాలాన్ని బట్టి మారుతూ ఉంటుందా? దయచేసి వివరించండి.

- - Why do you think that is?

అది ఎందుకు అని మీరు అనుకుంటున్నారు?

Storage of AFS

1. How do vendors store the [selected ASF] that are not sold? (e.g.: fridge/cupboard). Is there difference between largescale and small-scale vendors?

విక్రయించబడని లేదా అమ్మబడని [గుడ్డు/పాలు] విక్రేతలు ఎలా నిల్వ చేస్తారు? (ఉదా: ఫ్రిజ్/అలమరా). పెద్ద స్థాయి మరియు చిన్న-స్థాయి విక్రేతల మధ్య తేడా ఉందా?

1. What is the longest time vendors generally keep [selected ASF] that are not sold?

సాధారణంగా విక్రయించబడని [గుడ్డు/పాలు] పొడవైన విక్రేతలు ఏది ఉంచుతారు?

Price and governance of ASFs

1. Does the price vary depend on supplier, season, (size) or quality of [selected ASF]?

ధర అనేది సరఫరాదారుడు , కాలాన్ని బట్టి ,పరిమాణం లేదా [గుడ్డు/పాలు] యొక్క నాణ్యతపై ఆధారపడి ధర మారుతుందా?

- - Are there any other factors that affect the price you pay?

మీరు చెల్లించే ధరను ప్రభావితం చేసే ఇతర అంశాలు ఏమైనా ఉన్నాయా?

1. How do vendors set the price for the consumers? Prompts: vary depending on consumer, (size) or quality of [selected ASF] or regular customer

విక్రేతలు వినియోగదారుల కోసం ధరను ఎలా నిర్ణయిస్తారు? ప్రాంప్ట్‌లు: వినియోగదారుని పరిస్థితి , [గుడ్డు/పాలు] పరిమాణం లేదా నాణ్యత లేదా తరచుగా వచ్చే వినియోగదారుని (రెగ్యులర్ కన్స్యూమర్) బట్టి మారుతూ ఉంటాయి.

- How does this change throughout the year?

సంవత్సరం పొడవునా ఇది ఎలా మారుతుంది?

- - Are there other factors that affect price?

ధరను ప్రభావితం చేసే ఇతర అంశాలు ఏమైనా ఉన్నాయా?

1. Other than the cost of [selected ASF] what other costs are involved for vendors when trading [selected ASF]?

విక్రేతలు [గుడ్డు/పాలు] వ్యాపారం చేసేటప్పుడు విక్రేతలకు ఇతర ఖర్చులు ఏమైనా ఉంటాయి?

1. Who/which institutions can influence price of [selected ASF]?

ఎవరు/ఏ సంస్థలు [గుడ్డు/పాలు] ధరను ప్రభావితం చేస్తారు ?

- - How are these decisions made and what are the factors involved in such decisions?)

ఈ నిర్ణయాలు ఎలా తీసుకోబడ్డాయి మరియు అటువంటి నిర్ణయాలలో ఏఏ అంశాలు ఇమిడి ఉన్నాయి?

1. Are there any rules/ regulations on price of [selected ASF]?

[గుడ్డు/పాలు] ధరపై ఏవైనా నియమాలు/నిబంధనలు ఉన్నాయా?

1. Who/which institutions oversee developing and enforcing these regulations?

ఈ నిబంధనలను అభివృద్ధి చేయడం మరియు అమలు చేయడాన్ని ఎవరు/ఏ సంస్థలు పర్యవేక్షిస్తాయి?

1. Are these regulations effectively enforced?

ఈ నిబంధనలు సమర్థవంతంగా అమలవుతున్నాయా?

- - Why or why not?

ఎందుకు లేదా ఎందుకు కాదు?

1. What is the capacity of enforcers?

అమలు చేసేవారి సామర్థ్యం ఏమిటి?

- - How often are these regulations enforced?

ఈ నిబంధనలు ఎంత తరచుగా అమలు చేయబడతాయి?

- - At which level are these regulations enforced (i.e., is it only controlled for certain stakeholders?)

ఈ నిబంధనలు ఏ స్థాయిలో అమలు చేయబడతాయి? (అంటే, ఇది నిర్దిష్టముగా కొంతమంది వాటాదారుల కోసం మాత్రమే నియంత్రించబడుతుందా?

1. What are the consequences if a vendor does not comply with these regulations?

ఒకవేళ విక్రేత గనుక ఈ నిబంధనలను పాటించకపోతే ఎలాంటి పరిణామాలు ఉంటాయి ?

1. Do vendors generally have a written contract with suppliers?

సాధారణంగా విక్రేతలు , సరఫరాదారులతో ఏదైనా వ్రాతపూర్వక ఒప్పందాన్ని కలిగి ఉన్నారా?

1. Does it vary between small- and large-scale vendors?

ఈ ఒప్పందం అనేది చిన్న మరియు పెద్ద-స్థాయి విక్రేతల మధ్య ఏమైనా మారుతుందా?

1. What are the conditions often specified in such contract? *(Please do not ask hawkers as they may not have such arrangements)*

అటువంటి ఒప్పందంలో తరచుగా పేర్కొన బడిన షరతులు ఏమిటి? (దయచేసి హాకర్లను లేదా అనధికారిక విక్రయదారులను అడగవద్దు, ఎందుకంటే వారికి అలాంటి ఏర్పాట్లు ఉండకపోవచ్చు)

1. How do consumers pay vendors for [selected ASF]?

వినియోగదారులు [గుడ్లు/పాలు] కోసం విక్రేతలకు ఎలా చెల్లిస్తారు?

- 1. If vendors allow for different types of payment modalities, do these apply to any consumers, or some payments apply only to certain consumers? If some payments apply only to certain consumers, why is that?

విక్రేతలు వివిధ రకాల చెల్లింపు పద్ధతులను అనుమతించినట్లయితే, ఇవి అందరి వినియోగదారులకు వర్తిస్తాయా లేదా కొన్ని చెల్లింపులు నిర్దిష్ట వినియోగదారులకు మాత్రమే వర్తిస్తాయా? కొన్ని చెల్లింపులు నిర్దిష్ట వినియోగదారులకు మాత్రమే వర్తింపజేస్తే, అది ఎందుకు?

About their clients-Consumers

1. In your experience, what other characteristics do the consumers seek in vendors?

మీ అనుభవంలో వినియోగదారులు, విక్రేతలలో ఏ ఇతర లక్షణాలను కోరుకుంటారు?

1. Why do you think they avoid certain vendors?

వినియోగదారులు కొంతమంది విక్రేతలను ఎందుకు పట్టించుకోరు అని మీరు అనుకుంటున్నారు?

1. As we are interested in consumers based in slums, is there any specific supplier that supply [selected ASF] to vendors operating in slums?

మురికివాడల్లోని వినియోగదారులపై మాకు ఆసక్తి ఉన్నందున, మురికివాడల్లో పనిచేస్తున్న విక్రేతలకు [పాలు/గుడ్డు] సరఫరా చేసే నిర్దిష్ట సరఫరాదారు ఎవరైనా ఉన్నారా?

- - Why is it them specifically who supply them?

వాటిని ప్రత్యేకంగా ఎందుకు సరఫరా చేస్తారు?

**Semi-structured questionnaire for middlemen (traders/brokers/ wholesalers/transporters)**

General information - సాధారణ సమాచారం

*Selected ASF-Eggs and milk*

1. Who are major suppliers for [selected ASFs] and where are they based?

[గుడ్డు/పాలు] యొక్క ప్రధాన సరఫరాదారులు ఎవరు? మరియు వారు ఎక్కడ ఉన్నారు?

- 1. district and state

జిల్లా మరియు రాష్ట్రం?

- 1. type of farms: intensive/backyard…

పొలాల రకం: ఇంటెన్సివ్/పెరడు?

1. Do middlemen/wholesalers know details of the farming and management practices followed by their suppliers? (Prompts: rearing systems, biosecurity, farm structure, antibiotic usage)

మధ్యవర్తులు/హోల్‌సేల్ వ్యాపారులకు వారి సరఫరాదారులు అనుసరించే వ్యవసాయం మరియు నిర్వహణ పద్ధతుల వివరాలు తెలుసా? (ప్రాంప్ట్‌లు: పెంపకం వ్యవస్థలు, బయోసెక్యూరిటీ, వ్యవసాయ నిర్మాణం, యాంటీబయాటిక్ వాడకం)

1. Who are the major stakeholders, other than direct suppliers involved in providing middlemen/wholesalers with [selected ASFs]?

మధ్యవర్తులు మరియు హోల్ సేల్ వ్యాపారులకు [గుడ్లు/పాలు] అందించడంలో ప్రత్యక్ష సరఫరాదారులు కాకుండా ఇతర ప్రధాన లబ్దిదారుల ఎవరు?

- 1. And what role do they play? *(Can refer back to the diagram)*

మరియు వారు ఏ పాత్ర పోషిస్తారు? (రేఖాచిత్రాన్ని తిరిగి చూడవచ్చు)

Transportation - రవాణా

1. How are the [selected ASFs] transported to middlemen and wholesalers? (bike/motorbike, individual car, commercial van, lorry, cart, horse, etc.)

మధ్యవర్తులు మరియు హోల్ సేల్ వ్యాపారులకు [గుడ్డు/పాలు] సరుకు ఎలా రవాణా చేయబడుతుంది? (బైక్/మోటర్‌బైక్, వ్యక్తిగత కారు, వాణిజ్య వ్యాన్, లారీ, బండి, గుర్రం మొదలైనవి)

- 1. Please describe the conditions in which [selected ASFs] are transported (type of lorry, wagon, temperature)

దయచేసి [గుడ్డు/పాలు] రవాణా చేయబడే పరిస్థితులను వివరించండి (లారీ రకం, బండి, ఉష్ణోగ్రత)

Quality of ASF

1. How do middlemen and wholesalers evaluate the quality of [selected ASFs]?

మధ్యవర్తులు మరియు హోల్ సేల్ వ్యాపారులు [పాలు/గుడ్డు] నాణ్యతను ఎలా అంచనా వేస్తారు?

1. Is there a difference in quality of [selected ASFs] among different suppliers?

వివిధ సరఫరాదారుల మధ్య [గుడ్డు/పాలు] యొక్క నాణ్యతలో తేడా ఉందా?

- 1. If yes, what is the difference?

అవును అయితే, తేడా ఏమిటి?

- 1. If yes, why do you think the difference exists?

అవును అయితే, తేడా ఎందుకు ఉందని మీరు అనుకుంటున్నారు?

- 1. How do most people deal with poor quality [selected ASFs]? (e.g.: discard and feedback to supplier, sell it for lower price, sell it to different type of consumers)

చాలా మంది వ్యక్తులు నాణ్యత లేని [గుడ్లు/పాలు]తో ఎలా వ్యవహరిస్తారు? (ఉదా: పారవేయడం , సరఫరాదారుకు అభిప్రాయం చెప్పటం , తక్కువ ధరకు విక్రయించడం, వివిధ రకాల వినియోగదారులకు విక్రయించడం)

1. In the diagram, do products with different quality go through different channels?

రేఖాచిత్రంలో విభిన్న నాణ్యత కలిగిన ఉత్పత్తులు వేర్వేరు ఛానెల్‌ల ద్వారా వెళ్తాయా?

- 1. If yes, why do you think that is? What differentiates actors involved in trading good quality [Selected ASF] from actors involved in trading poor quality [Selected ASF]? (Prompts: Operation/business size, knowledge and expertise, geographical location, infrastructure and technology used, socio-economic status - income, ethnicity, gender, caste, landlessness, etc.)

అవును అయితే, అది ఎందుకు అని మీరు అనుకుంటున్నారు? మంచి నాణ్యత గల [పాలు/గుడ్డు] ట్రేడింగ్‌లో పాల్గొనే వ్యక్తులను నాసిరకం లేదా తక్కువ నాణ్యతతో కూడిన [పాలు/గుడ్డు] ట్రేడింగ్‌లో పాల్గొన్న వ్యక్తుల మధ్య తేడా ఏమిటి? (ప్రాంప్ట్‌లు: ఆపరేషన్/వ్యాపార పరిమాణం, జ్ఞానం, అవగాహనా మరియు నైపుణ్యం, భౌగోళిక స్థానం, ఉపయోగించిన మౌలిక సదుపాయాలు మరియు సాంకేతికత, సామాజిక-ఆర్థిక స్థితి - ఆదాయం, జాతి, లింగం, కులం, భూమిలేనితనం మొదలైనవి)

- 1. Can you describe these routes on the diagram?

మీరు ఈ మార్గాలను రేఖాచిత్రంలో వివరించగలరా?

1. *Dairy sector India only:* There are frequently news reports about milk sold in the country being adulterated. It is said nearly 75% of the milk is adulterated. What do you think about the issue? Where do you think adulteration happens (where geographically and where in the chain?)? How do you think they do it? What are the penalties if a supplier or middlemen is caught selling adulterated milk? Do you think those penalties are enough to discourage adulteration?

డెయిరీ సెక్టార్ - భారతదేశం: మన దేశంలో విక్రయించే పాలు కల్తీ అవుతున్నాయని తరచుగా వార్తలు వస్తున్నాయి. దాదాపు 75% పాలలో కల్తీ ఉందని చెబుతున్నారు. సమస్య గురించి మీరు ఏమనుకుంటున్నారు? కల్తీ ఎక్కడ జరుగుతుందని మీరు అనుకుంటున్నారు (భౌగోళికంగా ఎక్కడ మరియు గొలుసులో ఎక్కడ?)? వారు దీన్ని ఎలా చేస్తారని మీరు అనుకుంటున్నారు? కల్తీ పాలను విక్రయిస్తున్న సరఫరాదారు లేదా మధ్యవర్తులు పట్టుబడితే జరిమానాలు ఏమిటి? కల్తీని నిరోధించడానికి ఆ జరిమానాలు సరిపోతాయని మీరు అనుకుంటున్నారా?

1. Are the [selected ASFs] priced differently based on the quality?

[గుడ్డు/పాలు] యొక్క నాణ్యత ఆధారంగా వేర్వేరుగా ధర అనేది నిర్ణయించబడుతుందా?

1. Are there certification schemes that ensure particular product quality?

నిర్దిష్ట ఉత్పత్తి యొక్క నాణ్యతను నిర్ధారించే ధృవీకరణ పథకాలు ఉన్నాయా?

- 1. If yes, how easy is it to produce these high-quality [selected ASFs]?

అవును అయితే, ఈ అధిక-నాణ్యత కలిగిన [గుడ్డు/పాలు] ఉత్పత్తి చేయడం ఎంత సులభం?

- 1. If yes, how available are these high-quality [selected ASFs]?

అవును అయితే, ఈ అధిక-నాణ్యత గల [గుడ్డు/పాలు] ఎలా అందుబాటులో ఉన్నాయి?

- 1. If yes, how reliable/trustworthy is that certification?

అవును అయితే, ఆ సర్టిఫికేషన్ పథకం ఎంత విశ్వసనీయమైనది/నమ్మక మైనది?

- 1. If yes, what proportion of food producers do you think adhere with the scheme conditions?

అవును అయితే, ఆ స్కీమ్ షరతులకు కట్టుబడి ఉన్న ఆహార ఉత్పత్తిదారుల నిష్పత్తి ఎంత?

1. Does maintaining the quality of [selected ASFs] require investment?

[గుడ్డు/పాలు] యొక్క కాపాడుకోవడానికి పెట్టుబడి అవసరమా?

- 1. If so, what kind (e.g. machinery, trained staff, learning the know-how, etc.)?

అలా అయితే, ఏ రకమైన పెట్టుబడి (ఉదా. యంత్రాలు, శిక్షణ పొందిన సిబ్బంది, జ్ఞానాన్ని నేర్చుకోవడం మొదలైనవి) అవసరం?

1. In your view, what are the main barriers/challenges for improving the quality of [selected ASFs]?

మీ దృష్టిలో, [గుడ్డు/పాలు] నాణ్యతను మెరుగుపరచడానికి ప్రధాన అడ్డంకులు/సవాళ్లు ఏమిటి?

1. What do you think are new and/or exclusive opportunities for producers who are willing to invest in better quality?

మెరుగైన నాణ్యతతో పెట్టుబడి పెట్టడానికి సిద్ధంగా ఉన్న కంపెనీలకు గల కొత్త లేదా ఇతర ప్రత్యేకమైన అవకాశాలు ఏవి ఉన్నాయి అని మీరు ఏమనుకుంటున్నారు?

1. In the diagram, please describe the characteristics of people who buy or sell [selected ASFs] with different quality based on:

రేఖాచిత్రంలో, దయచేసి కింది వాటి ఆధారంగా విభిన్న నాణ్యతతో [గుడ్డు/పాలు] కొనుగోలు చేసే లేదా విక్రయించే వ్యక్తుల లక్షణాలను వివరించండి

- Operation/business size

పని /వ్యాపార పరిమాణం:

- Knowledge and expertise

జ్ఞానం మరియు నైపుణ్యం

- Seasonality - కాలానుగుణత
- Geographical location - భౌగోళిక ప్రదేశం :
- Infrastructure and technology used

మౌలిక సదుపాయాలు మరియు ఉపయోగించబడిన సాంకేతికత

- Socio-economic status (e.g. income, ethnicity, gender, caste, landlessness, etc.)

సామాజిక-ఆర్థిక స్థితి (ఉదా. ఆదాయం, జాతి, లింగం, కులం, భూమిలేనితనం మొదలైనవి

Storage - నిల్వ

1. How do middlemen and wholesalers store the [selected ASFs] that are not sold?

మధ్యవర్తులు మరియు హోల్ సేల్ వ్యాపారులు విక్రయించబడని [పాలు/గుడ్డు] ఎలా నిల్వ చేస్తారు

- 1. please describe all the storage facilities you have seen/owned (maintenance practices such as cleaning, maintenance costs, location of the building, capacity etc) – *may be* *not relevant for small middlemen*

*దయచేసి మీరు చూసిన/ఇతర యాజమాన్యంలో ఉన్న అన్ని నిల్వ సౌకర్యాలను వివరించండి (క్లీనింగ్, నిర్వహణ ఖర్చులు, భవనం యొక్క ప్రదేశం , సామర్థ్యం మొదలైనవి వంటి నిర్వహణ పద్ధతులు) - చిన్న మధ్యవర్తులకు సంబంధించినవి కాకపోవచ్చు?*

1. What do they do with [selected ASFs] that were not sold and are approaching their shelf life

విక్రయించబడని లేదా అమ్మబడని మరియు వారి షెల్ఫ్ జీవితాన్ని సమీపిస్తున్న [పాలు/గుడ్డు]తో వారు ఏమి చేస్తారు?

1. What is the longest time middlemen and wholesalers keep the [selected ASFs] that are not sold?

మధ్యవర్తులు మరియు హోల్ సేల్ వ్యాపారులు విక్రయించబడని [పాలు/గుడ్డు] ని ఎంత ఎక్కువ కాలం వరకు ఉంచుతారు?

Labelling *(eggs only)*

1. Is there any kind of labelling on the eggs?

గుడ్లపై ఏదైనా లేబులింగ్ లేదా ముద్ర ఉందా?

- 1. If yes, what information is provided on the label

అవును అయితే, లేబుల్‌ లేదా ముద్ర పై ఏ సమాచారం అందించబడింది?

- 1. Who does the labelling?

లేబులింగ్ ఎవరు చేస్తారు?

Price and governance - ధర మరియు పాలన

1. Does price vary depending upon supplier, season, size or quality of [selected ASFs]?

సరఫరాదారు రకం , సీజన్, పరిమాణం లేదా [పాలు/గుడ్డు] నాణ్యతను బట్టి ధర మారుతుందా?

- 1. What other factors affect price?

ఏ ఇతర అంశాలు ధరను ప్రభావితం చేస్తాయి?

1. Does the price vary depending on consumer, season, size or quality of [selected ASFs]?

వినియోగదారుని అభిరుచి , సీజన్, పరిమాణం లేదా [పాలు/గుడ్లు] నాణ్యతను బట్టి ధర మారుతుందా?

- 1. What other factors affect the price consumers pay? Other than the cost of [selected ASFs] what other costs are involved for you when trading [selected ASFs]? (e.g.: transportations, labour, tolls, taxes, commissions, license costs etc.)

వినియోగదారులు చెల్లించే ధరను ఏ ఇతర అంశాలు ప్రభావితం చేస్తాయి?

[పాలు/గుడ్డు] ఖరీదు మాత్రమే కాకుండా [పాలు/గుడ్లు] వ్యాపారం చేసేటప్పుడు మీకు ఏ ఇతర ఖర్చులు ఉంటాయి? (ఉదా: రవాణా, కార్మికులు, టోల్‌లు, పన్నులు, కమీషన్లు, లైసెన్స్ ఖర్చులు మొదలైనవి)

i. Can you estimate the percentage of each cost and how frequent are they?

మీరు ఖర్చుపెట్టె ప్రతి ఖర్చు శాతాన్ని అంచనా వేయగలరా? మరియు అవి ఎంత తరచుగా ఉంటాయి?

1. Do [selected ASFs] that are priced differently end up in different types of sales points? (e.g.: low priced reaches slums and high prices in big supermarkets)

[గుడ్డు/పాలు] ధరలు అనేవి వివిధ రకాల విక్రయ కేంద్రాలలో వేర్వేరుగ ముగుస్తుందా? (ఉదా: మురికివాడలులో తక్కువ ధర మరియు పెద్ద సూపర్ మార్కెట్‌లలో అధిక ధరలు)

- 1. If yes, why do you think that happens?

అవును అయితే, అలా ఎందుకు జరుగుతుందని మీరు అనుకుంటున్నారు?

1. Who/which institutions influence price?

ధరను ఎవరు/ఏ సంస్థలు ప్రభావితం చేస్తాయి?

- 1. How is this made?

అది ఎలా నిర్ణయింపబడుతుంది ?

1. Are there any rules/ regulations on price of [selected ASFs]?

[పాలు/గుడ్డు] ధరపై ఏవైనా నియమాలు/నిబంధనలు ఉన్నాయా?

1. Who/which institutions oversee developing and enforcing these regulations?

ఈ నిబంధనలను అభివృద్ధి చేయడం మరియు అమలు చేయడాన్ని ఎవరు/ఏ సంస్థలు పర్యవేక్షిస్తాయి?

1. Are these regulations effectively enforced?

ఈ నిబంధనలు సమర్థవంతంగా అమలు చేయబడుతున్నాయా?

- 1. Why or why not?

ఎందుకు లేదా ఎందుకు కాదు

1. Do you think it would be better if the government announce a uniform price for [selected ASFs]?

[పాలు/గుడ్డు]కి ప్రభుత్వం ఒకే ధరల విధానాన్ని ప్రకటిస్తే బాగుంటుందని మీరు భావిస్తున్నారా?

1. What are the consequences if you do not comply with these regulations?

మీరు ఈ నిబంధనలను పాటించకుంటే పరిణామాలు ఏమిటి?

1. In your opinion, in each of the networks (*point to the diagram)* identified,

మీ అభిప్రాయం ప్రకారం, (రేఖాచిత్రము ఆధారంగా) ప్రతి నెట్‌వర్క్‌లో గుర్తించబడిన ,

- 1. Who are the most influential people in the current production and distribution of [selected ASFs]?

ప్రస్తుతము [గుడ్డు/పాలు] యొక్క ఉత్పత్తి మరియు పంపిణీలో అత్యంత ప్రభావవంతమైన వ్యక్తులు ఎవరు? ( రైతు , పంపిణీదారుడు, వినియోగదారుడు, విక్రేత మొదలైనవారు )

- - 1. Why are they so influential?

వారు ఎందుకు అంత ప్రభావం చూపుతున్నారు?

- 1. Who are the weakest nodes in the current production and distribution network (i.e. the least influential)?

ప్రస్తుత ఉత్పత్తి మరియు పంపిణీ నెట్‌వర్క్‌లో బలహీనమైన వ్యవస్థలు ఎవరు (అంటే అతి తక్కువ ప్రభావవంతమైనవి)?

- 1. Who do you think earns the highest profit among all actors?

ఈ మొత్తం ఉత్పత్తి మరియు పంపిణి వ్యవస్థలో ఎవరు ఎక్కువ లాభం పొందుతారని మీరు అనుకుంటున్నారు?

- 1. Who earns the lowest profit among all actors?

ఈ మొత్తం ఉత్పత్తి మరియు పంపిణి వ్యవస్థలో ఎవరు తక్కువ లాభం పొందుతారని మీరు అనుకుంటున్నారు?

1. Are there any major association(s) that has/have a major influence on farmers, companies, consumers, etc.?

రైతులు, కంపెనీలు, వినియోగదారులు మొదలైన వాటిపై ప్రధాన ప్రభావం చూపే ఏదైనా ప్రధాన సంఘం(లు) ఉన్నాయా?

1. Please describe financial and contractual arrangements in the diagram drawn.

దయచేసి గీయబడిన రేఖాచిత్రంలో ఆర్థిక మరియు ఒప్పంద వివరాలను వివరించండి.

- 1. Is there any written agreement/contract with between different stakeholders?

వివిధ లబ్దిదారుల మధ్య ఏదైనా వ్రాతపూర్వక ఒప్పందం/ఒప్పందం ఉందా?

- 1. If yes, what does the contract dictate? *(Prompts: timing of the delivery, quantity, price, quality)*

అవును అయితే, ఒప్పందం ఏమి నిర్దేశిస్తుంది? (ప్రాంప్ట్‌లు: డెలివరీ సమయం, పరిమాణం, ధర, నాణ్యత)

1. Who normally operates using written contracts?

వ్రాతపూర్వక ఒప్పందాలను ఉపయోగించి సాధారణంగా ఎవరు ఆపరేట్/పని చేస్తారు?

- 1. Why? ఎందుకు
  2. How do the others then operate to reach an agreement?

ఒక ఒప్పందాన్ని చేరుకోవడానికి ఇతరులు ఎలా పని చేస్తారు?

1. How are payments normally done in this network (*point to the diagram)*?

ఈ మొత్తం నెట్‌వర్క్‌లో సాధారణంగా చెల్లింపులు ఎలా జరుగుతాయి (రేఖాచిత్రానికి పాయింట్)?

1. Are there different types of payment modalities within the network, do these apply to any consumers, or some payments apply only to certain consumers?

ఈ నెట్‌వర్క్‌లో వివిధ రకాల చెల్లింపు పద్ధతులు ఏమైనా ఉన్నాయా, ఇవి ఏ వినియోగదారులకైనా వర్తిస్తాయా లేదా కొన్ని చెల్లింపులు కొంతమంది నిర్దిష్ట వినియోగదారులకు మాత్రమే వర్తిస్తాయా?

- - 1. If some payments apply only to certain consumers, why is that?

కొన్ని రకాల చెల్లింపులు కొంతమంది నిర్దిష్ట వినియోగదారులకు మాత్రమే వర్తింపజేస్తే, అది ఎందుకు?

1. How do people get the capital to operate in this network?

ఈ మొత్తం వ్యవస్థ నెట్‌వర్క్‌లో పనిచేయడానికి వ్యక్తులు తమ యొక్క మూలధనాన్ని ఎలా పొందుతారు?

- 1. Are there any financial institution or person supporting them?

వారికి సహకరించే ఆర్థిక సంస్థ లేదా వ్యక్తి ఎవరైనా ఉన్నారా?

- - 1. If so, please explain

అలా అయితే, దయచేసి వివరించండి

- 1. Are there any government subsidies that middlemen and wholesalers can access?

మధ్యవర్తులు మరియు హోల్ సేల్ వ్యాపారులు పొందగలిగే ప్రభుత్వ సబ్సిడీలు ఏమైనా ఉన్నాయా?

- - 1. Please explain who provides it and criteria to be eligible

దయచేసి దానిని ఎవరు అందిస్తారో మరియు అర్హత పొందేందుకు గల ప్రమాణాలను వివరించండి

**Semi-structured questionnaire for farmers (producers/dairy cooperatives/ farmer association/ poultry breeding association/ breeders)**

**Poultry farmers**

General information - సాధారణ సమాచారం

1. Who are the main suppliers for birds in Hyderabad and where are they based?

హైదరాబాద్‌లో పక్షులకు లేదా కోళ్లకు ప్రధాన సరఫరాదారులు ఎవరు మరియు అవి ఎక్కడ ఉన్నాయి?

- 1. district and state - జిల్లా మరియు రాష్ట్రం?

1. Who are the popular suppliers for bird feed and where are they based?

పక్షి (కోళ్ళు) మేత కోసం ప్రముఖ సరఫరాదారులు ఎవరు మరియు వారు ఎక్కడ ఉన్నారు?

- 1. district and state - జిల్లా మరియు రాష్ట్రం?

1. Who are the major stakeholders, other than the company providing birds and feed involved in poultry business?

పౌల్ట్రీ వ్యాపారంలో పాలుపంచుకున్న పక్షులు (కోళ్లు) మరియు వాటి దాణాను అందించే సంస్థ కాకుండా ప్రధాన వాటాదారులు ఇంకా ఎవరు ఉన్నారు ?

- 1. And what are their roles? *(Can refer back to the diagram)*

మరియు వారి పాత్రలు ఏమిటి? (రేఖాచిత్రాన్ని తిరిగి సూచించవచ్చు

1. Who are the customers of layer farmers? *(can refer back to the diagram)*

లేయర్ (గుడ్లు) రైతుల కస్టమర్లు ఎవరు? (రేఖాచిత్రాన్ని తిరిగి చూడవచ్చు)

Labelling (eggs only)

1. Do farmers add any labelling to the eggs prior to selling them?

రైతులు గుడ్లను విక్రయించే ముందు వాటికి ఏదైనా లేబులింగ్‌ని జోడించారా?

- 1. If yes, what information is provided on the label (eg: expiry date)

అవును అయితే, లేబుల్‌పై ఏ సమాచారం అందించబడింది (ఉదా: గడువు తేదీ)

**Dairy farmers**

General information - సాధారణ సమాచారం

1. Where do dairy farmers generally purchase dairy cows from?

సాధారణంగా పాడి రైతులు పాడి ఆవులను ఎక్కడ నుండి కొనుగోలు చేస్తారు?

- 1. Name of the markets, district, and state

మార్కెట్లు, జిల్లా మరియు రాష్ట్రం పేరు

1. Who are the popular suppliers for cow feed and where are they based?

ఆవు మేత కోసం ప్రముఖ సరఫరాదారులు ఎవరు మరియు వారు ఎక్కడ ఉన్నారు?

- 1. district and state - జిల్లా మరియు రాష్ట్రం?

1. Who are the major stakeholders, other than the company providing birds and feed involved in dairy business?

డైరీ వ్యాపారంలో పాలుపంచుకున్న పక్షులు (కోళ్లు) మరియు దాణాను అందించే సంస్థ కాకుండా ప్రధాన వాటాదారులు ఎవరు?

- 1. And what are their roles? *(can refer back to the diagram)*

మరియు వారి పాత్రలు ఏమిటి? (రేఖాచిత్రాన్ని తిరిగి సూచించవచ్చు

1. Who are the customers of dairy farmers? *(can refer back to the diagram)*

పాడి రైతుల కస్టమర్లు ఎవరు? (రేఖాచిత్రాన్ని తిరిగి చూడవచ్చు)

*The remaining sections are common for all type of farmers.*

Transportation - రవాణా

1. How do farmers and their customers transport [selected ASFs]? *(bike/motorbike, individual car, commercial van, lorry)*

రైతులు మరియు వారి వినియోగదారులు [గుడ్డు/పాలు] ఎలా రవాణా చేస్తారు? (బైక్/మోటార్‌బైక్, వ్యక్తిగత కారు, వాణిజ్య వ్యాన్, లారీ)

1. Please describe the conditions in which [selected ASFs] are transported? *(type of lorries, temperature)*

దయచేసి [గుడ్డు/పాలు] రవాణా చేయబడే పరిస్థితులను వివరించండి (లారీల రకం, ఉష్ణోగ్రత)

Storage

1. How do farmers store the [selected ASFs] that are not sold?

రైతులు విక్రయించబడని [గుడ్డు/పాలు] ఎలా నిల్వ చేస్తారు?

- 1. Please describe the type of storage facilities used by farmers you have seen from your experience, or you have used (ownership of the building, maintenance practices such as cleaning, maintenance costs, location of the building, capacity etc) – may be *not relevant for small farmers*

*దయచేసి మీ అనుభవం నుండి లేదా మీరు చూసిన లేక మీకు తెలిసిన రైతులు ఉపయోగించిన నిల్వ సౌకర్యాల రకాన్ని వివరించండి లేదా మీరు ఉపయోగించిన (భవనం యొక్క యాజమాన్యం, శుభ్రపరచడం, నిర్వహణ ఖర్చులు, భవనం యొక్క స్థానం, సామర్థ్యం మొదలైనవి వంటి నిర్వహణ పద్ధతులు) – చిన్న రైతులు కి సంబంధించినవి కాకపోవచ్చు*

1. Do you think currently farmers have facilities that allow you to store [selected ASFs]?

ప్రస్తుతం రైతులు [పాలు/గుడ్డు] నిల్వ చేసుకునేందుకు వీలు కల్పించే సౌకర్యాలు ఉన్నాయని మీరు భావిస్తున్నారా?

1. *Poultry farmers only*: What do farmers do with eggs that they could not sell and are approaching their shelf life?

పౌల్ట్రీ రైతులు మాత్రమే: రైతులు విక్రయించలేని మరియు వారి గడువు కాలానికి చేరువైన గుడ్లను ఏమి చేస్తారు?.

Quality of ASF

1. How do farmers evaluate the quality of [selected ASFs]? What do they do with poor quality [selected ASFs]? (example: sell it cheaper, sell it to specific consumers, discard etc.)

రైతులు [పాలు/గుడ్డు] నాణ్యతను ఎలా అంచనా వేస్తారు? నాణ్యత లేని [గుడ్డు/పాలు]తో వారు ఏమి చేస్తారు? (ఉదాహరణ: చౌకగా విక్రయించడం, నిర్దిష్ట వినియోగదారులకు విక్రయించడం, విస్మరించండి మొదలైనవి)

1. *Dairy farmers only:* What type of tests are used by milk collectors and/or milk collecting centres to test the quality of milk? What happens to the milk identified as poor quality? Do the criteria for quality vary between different centres and collectors?

పాడి రైతులు కి మాత్రమే: పాల నాణ్యతను పరీక్షించడానికి పాల సేకరణదారులు మరియు/లేదా పాల సేకరణ కేంద్రాలు ఏ రకమైన పరీక్షలను ఉపయోగిస్తాయి? నాణ్యత లేని పాలుగా గుర్తించిన పాలు ఏమయ్యాయి? వివిధ పాల సేకరణ కేంద్రాలు మరియు సేకరించే వాళ్ళ మధ్య నాణ్యత ప్రమాణాలు మారుతున్నాయా?

1. *Dairy farmers India only:* There are frequently news reports about milk sold in the country being adulterated. It is said nearly 75% of the milk is adulterated. What do you think about the issue? Where do you think adulteration happens? How do you think they do it?

పాడి రైతులు భారతదేశంలో మాత్రమే: దేశంలో విక్రయించే పాలు కల్తీ అవుతున్నాయని తరచుగా వార్తలు వస్తున్నాయి. దాదాపు 75% పాలలో కల్తీ ఉందని చెబుతున్నారు. సమస్య గురించి మీరు ఏమనుకుంటున్నారు? కల్తీ ఎక్కడ జరుగుతుందని మీరు అనుకుంటున్నారు? వారు దీన్ని ఎలా చేస్తారని మీరు అనుకుంటున్నారు?

1. Are there certification schemes that ensure product quality?

ఉత్పత్తి నాణ్యతను నిర్ధారించే ధృవీకరణ పథకాలు ఉన్నాయా?

- 1. If yes, how easy is it to produce these high-quality [selected ASFs]?

అవును అయితే, ఈ అధిక-నాణ్యత కలిగిన [గుడ్డు/పాలు] ఉత్పత్తి చేయడం ఎంత సులభం?

- 1. If yes, how available are these high-quality [selected ASFs] ?

అవును అయితే, ఈ అధిక-నాణ్యత కలిగిన [గుడ్డు/పాలు] ఎలా అందుబాటులో ఉన్నాయి?

- 1. What proportion of farmers do you think are part of the scheme? What proportion do you think adhere to the conditions of the scheme?

ఈ పథకంలో ఎంత మంది రైతులు భాగస్వాములని మీరు అనుకుంటున్నారు? మరియు ఈ పథకం యొక్క షరతులకు ఎంతవరకు కట్టుబడి ఉందని మీరు అనుకుంటున్నారు?

1. Does producing good quality [selected ASFs] and maintaining the quality of [selected ASFs] require investment? If so, what kind (e.g., machinery, trained staff, learning the know-how, etc.)?

మంచి నాణ్యమైన [గుడ్డు/పాలు] ఉత్పత్తి చేయడానికి మరియు కాపాడుకోవడానికి ఏదైనా పెట్టుబడి అవసరమా? అలా అయితే, ఏ రకమైన పెట్టుబడి అవసరం (ఉదా., యంత్రాలు, శిక్షణ పొందిన సిబ్బంది, జ్ఞానాన్ని నేర్చుకోవడం మొదలైనవి)?

1. In your view, what are the main barriers/challenges for improving the quality of [selected ASFs]?

మీ దృష్టిలో, [గుడ్డు/పాలు] నాణ్యతను మెరుగుపరచడానికి ప్రధాన అడ్డంకులు/సవాళ్లు ఏమిటి?

1. What do you think are new and/or exclusive opportunities for producers who are willing to invest in better quality?

మెరుగైన నాణ్యతతో పెట్టుబడి పెట్టడానికి సిద్ధంగా ఉన్న ఉత్పత్తిదారులకు కొత్త మరియు/లేదా ప్రత్యేకమైన అవకాశాలు ఏమిటి అని మీరు ఏమనుకుంటున్నారు?

1. What are the things farmers do on farms to ensure good quality [selected ASFs]?

నాణ్యమైన [గుడ్డు/పాలు] ఉండేలా రైతులు వ్యవసాయంలో ఏమి చేస్తారు?

1. In the diagram, please describe the characteristics of people who buy or sell [selected ASFs] with different quality based on:

రేఖాచిత్రం ఆధారంగా విభిన్న నాణ్యతతో [గుడ్డు/పాలు] కొనుగోలు చేసే లేదా విక్రయించే వ్యక్తుల మధ్య లక్షణాలను వివరించండి:

- Operation/business size

పని /వ్యాపార పరిమాణం:

- Knowledge and expertise

జ్ఞానం మరియు నైపుణ్యం

- Seasonality - కాలానుగుణత
- Geographical location - భౌగోళిక ప్రదేశం :
- Infrastructure and technology used

మౌలిక సదుపాయాలు మరియు ఉపయోగించబడిన సాంకేతికత

- Socio-economic status (e.g. income, ethnicity, gender, caste, landlessness, etc.)

సామాజిక-ఆర్థిక స్థితి (ఉదా. ఆదాయం, జాతి, లింగం, కులం, భూమిలేనితనం మొదలైనవి

Price and governance

1. Does the price farmers charge vary depending on customer, season, size or quality of [selected ASFs]?
2. వినియోగదారు రకం , సీజన్, పరిమాణం లేదా [పాలు/గుడ్డు] నాణ్యతను బట్టి ధర మారుతుందా?
3. What are the other factors that affect price they charge? / Other than the cost of animals and feed what other costs are involved for you when trading [selected ASFs]? (e.g.: transportations, labour, tolls, taxes, commissions, license costs etc.)

వారు వసూలు చేసే ధరను ప్రభావితం చేసే ఇతర అంశాలు ఏమిటి? / జంతువులు మరియు ఫీడ్ ఖర్చు కాకుండా [పాలు/గుడ్డు] వ్యాపారం చేసేటప్పుడు మీకు ఏ ఇతర ఖర్చులు ఉంటాయి? (ఉదా: రవాణా, కార్మికులు, టోల్‌లు, పన్నులు, కమీషన్లు, లైసెన్స్ ఖర్చులు మొదలైనవి)

1. Can you estimate the percentage of each cost and how frequent are they?

మీరు ఖర్చుపెట్ట్టె ప్రతి దాని యొక్క శాతాన్ని అంచనా వేయగలరా మరియు అవి ఎంత తరచుగా ఉంటాయి?

1. Do [selected ASFs] that are price differently end up in different type sales points? (e.g.: low priced reaches slums and high prices in big supermarkets)
2. [గుడ్డు/పాలు] ధరలు అనేవి వివిధ రకాల విక్రయ కేంద్రాలలో వేర్వేరుగ ముగుస్తుందా? (ఉదా: మురికివాడలులో తక్కువ ధర మరియు పెద్ద సూపర్ మార్కెట్‌లలో అధిక ధరలు)

a. If yes, why do you think that happens? అవును అయితే, అలా ఎందుకు జరుగుతుందని మీరు అనుకుంటున్నారు?

1. Who/which institutions are in charge of deciding on price?

ధరపై నిర్ణయం తీసుకునే బాధ్యత ఎవరి/ఏ సంస్థలకు ఉంది?

- 1. How is this made?

ఇది ఎలా నిర్ణయించబడింది?

1. Are there any rules/ regulations on price of [selected ASFs]?

[గుడ్డు/పాలు] ధరపై ఏవైనా నియమాలు/నిబంధనలు ఉన్నాయా?

1. Who/which institutions are in charge of developing and enforcing these regulations?

ఈ నిబంధనలను అభివృద్ధి చేయడానికి మరియు అమలు చేయడానికి ఎవరు/ఏ సంస్థలు బాధ్యత వహిస్తాయి?

1. Are these regulations effectively enforced?

ఈ నిబంధనలు సమర్థవంతంగా అమలు చేయబడుతున్నాయా?

- 1. Why or why not?

ఎందుకు లేదా ఎందుకు కాదు?

1. What is the capacity of enforcers?

అమలు చేసేవారి సామర్థ్యం ఏమిటి?

- 1. How often are these regulations actually enforced?

వాస్తవానికి ఈ నిబంధనలు ఎంత తరచుగా అమలు చేయబడతాయి?

- 1. At which level are these regulations actually enforced (i.e. is it only controlled for certain stakeholders?

ఈ నిబంధనలు వాస్తవానికి ఏ స్థాయిలో అమలు చేయబడతాయి (అంటే ఇది నిర్దిష్ట వాటాదారుల కోసం మాత్రమే నియంత్రించబడుతుందా?

1. Do you think it would be better if the government announced a uniform price?

ప్రభుత్వం ధరను ఒకే విధంగా ప్రకటిస్తే బాగుంటుందని భావిస్తున్నారా?

1. What are the consequences if you do not comply with these regulations?

మీరు ఈ నిబంధనలను పాటించకుంటే పరిణామాలు ఏమిటి?

1. In your opinion, in each of the networks (*point to the diagram)* identified, who are the most influential people in the current production and distribution of [selected ASFs]?

మీ అభిప్రాయం ప్రకారం, గుర్తించబడిన ప్రతి నెట్‌వర్క్‌లలో లేదా ఈ మొత్తం వ్యవహారంలో ప్రస్తుత [గుడ్డు/పాలు] ఉత్పత్తి మరియు పంపిణీలో అత్యంత ప్రభావవంతమైన వ్యక్తులు ఎవరు? (రేఖాచిత్రాన్ని సూచించండి)

- 1. Why are they so influential?

వారు ఎందుకు అంత ప్రభావం చూపుతున్నారు?

1. Who do you think earns the highest profit among all actors?

ఈ మొత్తం గొలుసుకట్టు వ్యవహారం లో ఎవరు ఎక్కువ లాభం పొందుతారని మీరు అనుకుంటున్నారు?

1. Who earns the lowest profit among all actors?

ఈ మొత్తం గొలుసుకట్టు వ్యవహారం లో ఎవరు తక్కువ లాభం పొందుతారని మీరు అనుకుంటున్నారు?

1. Who has the knowledge about poultry diseases and health risks?

పౌల్ట్రీ రంగం లో వచ్చే వ్యాధులు మరియు ఆరోగ్య ప్రమాదాల గురించి ఎవరికి తెలుసు?

1. Are there any major association(s) that has/have a major influence on farmers, companies, consumers, etc.?

రైతులు, కంపెనీలు, వినియోగదారులు మొదలైన వాటిపై ప్రధాన ప్రభావం చూపే ఏదైనా ప్రధాన సంఘం(లు) ఉన్నాయా?

1. Please describe financial and contractual arrangements in the diagram drawn.

దయచేసి గీసిన రేఖాచిత్రంలో ఆర్థిక మరియు ఒప్పంద నకు సంబందించిన ఏర్పాట్లను/వివరాలను వివరించండి.

1. Do you have any written agreement/contract with your suppliers or consumers?

మీరు మీ సరఫరాదారులు లేదా వినియోగదారులతో ఏదైనా వ్రాతపూర్వక ఒప్పందం/ఒప్పందాన్ని కలిగి ఉన్నారా?

- 1. If yes, what does the contract dictate? *(prompts : timing of the delivery, quantity, price, quality)*

అవును అయితే, ఒప్పందం ఏమి నిర్దేశిస్తుంది? ఏ ఏ అంశాలు (ప్రాంప్ట్‌లు: డెలివరీ సమయం, పరిమాణం, ధర, నాణ్యత)

1. Who normally operates using written contracts?

సాధారణంగా వ్రాతపూర్వక ఒప్పందాలను ఉపయోగించి ఎవరు పని చేస్తారు?

- 1. Why? ఎందుకు
  2. How do the others then operate to reach an agreement?

ఒక ఒప్పందాన్ని చేరుకోవడానికి ఇతరులు ఎలా పని చేస్తారు?

1. How do people pay farmers for [selected ASFs]?

[గుడ్డు/పాలు] కోసం ప్రజలు రైతులకు ఎలా చెల్లిస్తారు?

- 1. If they allow for different types of payment modalities, do these apply to any consumers, or some payments apply only to certain consumers?

వారు వివిధ రకాల చెల్లింపు పద్ధతులను అనుమతిస్తే, ఇవి వినియోగదారులకు వర్తిస్తాయా లేదా కొన్ని చెల్లింపులు కొంత మంది నిర్దిష్ట వినియోగదారులకు మాత్రమే వర్తిస్తాయా?

- - 1. If some payments apply only to certain consumers, why is that?

కొన్ని చెల్లింపులు నిర్దిష్టముగా కొంత మంది వినియోగదారులకు మాత్రమే వర్తింపజేస్తే, అది ఎందుకు?

- 1. Do farmers operate on credit system?

రైతులు క్రెడిట్ సిస్టమ్‌లో లేదా అరువు పద్దతి లో పనిచేస్తున్నారా?

1. Is there any government subsidies that farmers can access to support their business?

రైతులు తమ వ్యాపారానికి మద్దతు ఇవ్వడానికి ప్రభుత్వ నుండి ఏమైనా రాయితీలు ఉన్నాయా?

- - 1. Please explain who provides it and criteria to be eligible

దయచేసి దానిని ఎవరు అందిస్తారో మరియు అర్హత పొందేందుకు గల ప్రమాణాలను వివరించండి

**Semi-structured questionnaire for official of egg industry associations**

**English interviews only**

General questions

1. Could you tell us about your experience and role in the [egg industry association]?
2. Can you explain to us how the [egg industry association] operate?
3. In your understanding, what is the role of [egg industry association] in poultry sector? Has the role of [egg industry association] changed since its inception and if yes, how?
4. How many farmers are members in the [egg industry association]? What are the criteria for the membership in the [egg industry association]?
5. Can you categorise the farmers (small, medium, and large) according to poultry numbers/ business volume?

Specific questions:

1. Who are the main actors who influence how the chains are shaped?
2. Do you have studies on the profit margins of different actors in the egg value chain? We are interested in knowing what the distribution is of the margins in the chains.
   - 1. If such data are not available: In your experience, which actors in the value chains benefit the most and why?
3. In your opinion, in each of the networks (*point to the diagram)* identified, who are the most influential people in the current production and distribution of eggs?
   1. Why are they so influential?
4. Who do you think earns the highest profit among all actors?
5. Who earns the lowest profit among all actors?
6. Who has the knowledge about poultry diseases and health risks?
7. Are there any major association(s) apart from [egg industry associations] that has a major influence on farmers, companies, consumers, etc.?
8. Please describe financial and contractual arrangements in the diagram drawn.
9. Do eggs that are price differently end up in different type sales points? (eg: low priced reaches slums and high prices in big supermarkets)
   1. If yes, why do you think that happens and what could be done to improve the situation for stunted children?

Prices and the role of eggs

1. We have some specific questions on your operations:
   1. What are the criteria for setting the egg price and who decides on the price (and how often is that done)? (prompts: economic analysis, real-time data, discussions with stakeholders including farmers, etc)
   2. Does the price of egg change depend on customer, season, size or quality of eggs?
   3. What are the other factors that affect price they charge? Other than the cost of animals and feed what other costs are involved for you when trading eggs? (e.g.: transportations, labour, tolls, taxes, commissions, license costs etc.)
   4. Can you estimate the percentage of each cost and how frequent are they?
   5. Are there any rules/ regulations on price decision for [egg industry associations] which institutions oversee developing and enforcing these regulations? Do you think it would be better if the government announced a uniform price?
   6. If there is a disease outbreak/ quality issues- what means do you have to support the farmers?
   7. How does [egg industry association] link or collaborate with other institutions including industry organisations, government or NGOs? (prompts: farmer associations, ministries, insurance companies, pharmaceutical companies, etc.)
   8. What is [egg industry association]’s role or influence when it comes to policy setting?
   9. Does [egg industry association] communicate with farmers and take on board their input? If yes, how does that work?
   10. Does [egg industry association] have activities in place to enhance food safety and if yes, what are they?

Suppliers

1. Who are the main suppliers for birds in Hyderabad and where are they based?
   1. district and state- list top 5 companies and their share in the market
2. Who are the popular suppliers for bird feed and where are they based? Is there any contract farming for layers?
   1. district and state

Storage of eggs

1. How do farmers store eggs that are not collected every day and eggs that are not sold?
   1. Please describe the type of storage facilities used by farmers you have seen from your experience, or you have used (ownership of the building, maintenance practices such as cleaning, maintenance costs, location of the building, capacity etc)?
   2. Does storage vary between small, medium and large farmers?
   3. What do farmers do with eggs that they could not sell and are approaching their shelf life?

Quality of ASF (egg)

1. According to [egg industry association], what constitutes a good quality egg and how is this assessed? *(Prompt: Taste, size, chemical and microbiological hazards, shell, etc.).*
2. What would you say is the egg quality overall in India and are there certain quality aspects that [egg industry associations] would like to improve? If yes, what are they?
3. What happens with poor quality eggs and does [egg industry association] have any influence over what happens with them? (e.g.: sell it cheaper, sell it to specific consumers, discard
   1. Does the [egg industry association] provide information on egg safety, how to discard, etc)
4. Are there certification schemes that ensure product quality?
   1. Are there any norms from [egg industry associations] that farmers can follow? If yes, what are they?
   2. Are you aware of any (other) certification schemes?
   3. If yes, how easy is it to produce these high-quality eggs?
   4. If yes, how available are these high-quality eggs?
   5. If yes, what proportion of farmers do you think are part of high-quality egg production?
   6. Do the high-quality egg production require any investment like machineries, technical staff, capacity/training?
5. In your view, what are the main barriers/challenges for improving the quality of eggs?
6. What do you think are new and/or exclusive opportunities for producers who are willing to invest in better quality?
7. What are the things farmers do on farm to ensure good quality eggs?

Contracts

1. Do you have any written agreement/contract with your members?
   1. If yes, what does the contract dictate? (prompts: timing of the delivery, quantity, price, quality)
2. Who normally operates using written contracts?
   1. Why?
   2. How do the others then operate to reach an agreement?
3. How do companies/ traders pay farmers for eggs?
   1. If they allow for different types of payment modalities, do these apply to any consumers, or some payments apply only to certain consumers?
      1. If some payments apply only to certain consumers, why is that?
   2. Do farmers operate on credit system?
4. Are there any government subsidies that farmers can access to support their business?
   - 1. Please explain who provides it and criteria to be eligible

*End of interview – thank the informants for their time and participation*
